# Nonlinear Quantum Effects Drive Grotthuss Proton Conduction in Structured Electrolytes Based on Deep Eutectic Solvents

**DOI:** 10.1002/advs.77781

**Published:** 2026-10-08

**Authors:** Benworth B. Hansen, Michael S. Chen, Giselle de A. L. e Souza, Kaylie Glynn, Alan Robledo, Emilia Pelegano‐Titmuss, Katherine Hightower, Miguel Muñoz, Rathiesh Pandian, Clemens Burda, Aikaterini Sanida, Mark Dadmun, Burcu E. Gurkan, Steve G. Greenbaum, Mark E. Tuckerman, Joshua R. Sangoro

**Affiliations:** ^1^ William G. Lowrie Department of Chemical and Biomolecular Engineering The Ohio State University Columbus Ohio USA; ^2^ Department of Chemistry New York University New York New York USA; ^3^ Simons Center for Computational Physical Chemistry New York University New York New York USA; ^4^ Department of Physics and Astronomy Hunter College of City University of New York New York New York USA; ^5^ Department of Chemical and Biomolecular Engineering Case Western Reserve University Cleveland Ohio USA; ^6^ Department of Chemistry University of Tennessee Knoxville Tennessee USA; ^7^ Department of Physics New York University New York New York USA; ^8^ Courant Institute of Mathematical Sciences New York University New York New York USA; ^9^ NYU‐ECNU Center for Computational Chemistry at NYU Shanghai Shanghai China

**Keywords:** deep eutectic solvents, diffusion coefficient, Grotthuss proton conduction, machine‐learning ab initio molecular dynamics simulations, path integrals method, proton transport

## Abstract

Structured electrolytes based on deep eutectic solvents (DESs) face electrochemical limitations of vehicular diffusion due to high viscosities often leading to low conductivities. Here, we demonstrate that the inverse relationship between viscosity and conductivity in DESs can be circumvented by leveraging their hydrogen bonding networks for Grotthuss proton transport. Using DESs comprising imidazole and levulinic acid, we show that the existence of extended hydrogen‐bonded chains coupled with rapid reorientation of protonated imidazoles result in enhanced Grotthuss proton diffusion in these concentrated hydrogen‐bonded electrolyte systems. Key machine‐learning driven simulations – crucially incorporating nuclear quantum effects (NQEs) – validate our experimental findings, which classical simulations fail to capture. This study demonstrates that NQEs promote imidazole protonation, catalyze the hydrogen‐bonded chain formation, and influence chain reorientation, resulting in enhanced dynamics surpassing classical predictions in structured electrolytes. While the conclusions are drawn here for a particular imidazole/acid system, they are expected to apply broadly to protic DESs that support chain‐like structures and exploit Grotthuss diffusion as the primary charge‐transport mechanism.

## Graphical abstract

We demonstrate that the inverse relationship between viscosity and conductivity in structured electrolytes can be circumvented by leveraging their hydrogen bonding networks for Grotthuss proton transport. Using a mixture of imidazole and levulinic acid, we show that the existence of extended hydrogen‐bonded chains that can rapidly reorient result in enhanced Grotthuss proton diffusion in these concentrated hydrogen‐bonded electrolyte systems. AI‐driven simulations demonstrate that nuclear quantum effects promote imidazole protonation, catalyze the hydrogen‐bonded chain formation, and influence chain reorientation, resulting in enhanced dynamics surpassing classical predictions in structured electrolytes.

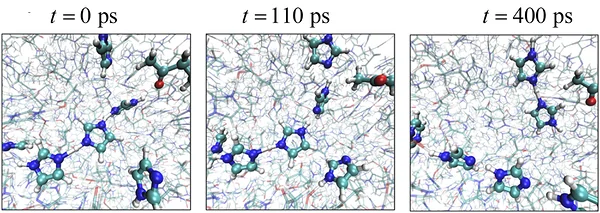

## 1 Introduction

The development of advanced structured electrolytes, engineered for rapid proton transport and proton‐coupled electron transfer (PCET) reactions [1, 2, 3], is frequently constrained by limitations inherent to vehicular diffusion mechanisms [4, 5, 6]. These mechanisms depend on the physical transport of a proton carrier, which restricts the availability of reactants at the electrochemical interface. However, an alternative and significantly faster pathway for proton transport is available via the *Grotthuss mechanism* (GM) [7, 8]. The GM, also known as the *structural diffusion mechanism*, facilitates proton conduction via a cooperative process, often likened to a Newton's cradle or fire bucket brigade, where protons “hop” sequentially along a network of hydrogen‐bonded molecules [9, 10, 11, 12, 13, 14]. This cascading transfer occurs on a timescale of tenths of a picosecond, a rate approximately 100 times faster than the molecular reorientation required for vehicular transport [11]. As such, materials that exhibit the GM can substantially accelerate proton transport.

In order to harness this phenomenon, recent efforts have focused on integrating the GM into novel electrolytes by designing compounds with amphoteric, proton‐donating functionalities [12, 14, 15, 16, 17, 18, 19, 20, 21, 22, 23, 24, 25, 26, 27, 28]. Pioneering work, such as that by Noda et al. [12] and Yaghini et al. [25, 26] has successfully demonstrated this principle by incorporating imidazole into ionic liquids (ILs). Their findings confirm that the resulting materials exhibit proton conduction through both Grotthuss and vehicular pathways. However, the next step should constitute an attempt to integrate the GM into systems predominantly composed of hydrogen bond donors and acceptors, such as deep eutectic solvents (DESs) [29, 30, 31]. The successful implementation of this strategy would yield a new class of advanced, structured electrolytes, which we term “concentrated hydrogen‐bonded electrolytes” (CoHBEs), that feature the many potential advantages of DESs [32, 33].

In the study of charge transport in electrolytes, a common assumption – which will herein serve as a working “null” hypothesis – is that macroscopic properties such as viscosity and conductivity are primarily or solely governed by vehicular diffusion [34]. For example, under this assumption, diffusion rates are viewed as surrogate measures of viscosity and packing, since the movement of proton‐carrying molecules heavily relies on the flexibility afforded by local cooperativity. However, since Grotthuss diffusion primarily depends on the formation of local HBNs, significantly faster structural diffusion can occur in a fashion that bypasses the aforementioned relationship.

To develop novel structured electrolytes that favor proton transport via an emergent GM, the resulting systems must a) promote extended hydrogen bonding networks (HBNs), and b) provide a source of surplus protons. While the formation of a robust hydrogen‐bonding network (HBN) is an intrinsic feature of deep eutectic solvents (DESs) [29], our approach must incorporate additional design criteria to control aspects of the network's topology. To achieve this, we stipulate that both DES components should be amphoteric in order to form extended H‐bond networks, rather than local pairing and/or micelle‐like clustering. Because we also wish to promote linear, chain‐like H‐bonding structures, we looked for compounds that possess roughly 2‐fold symmetry about their principal proton exchange sites. Finally, for the specific purposes of creating a general model system, we added further stipulations of compounds that would form a DES with a wide range of liquid compositions and that possess minimal extraneous functional groups to reduce effects from steric hindrance [28]. From our study, we aim to extract general mechanisms that govern broader classes of protic DESs and expose the connection between viscosity and conductivity in systems dominated by Grotthuss diffusion.

To this end, we present a model CoHBE formed from imidazole and levulinic acid. Imidazole (Im) (C_3_H_4_N_2_, *T*_m_ ≈ 363 K) is known to form extended hydrogen‐bonded structures in the presence of excess protons that enable the GM [14]. However, it does not easily autoionize [35, 36, 37] and thus requires an external source of acidic, ionizable protons to form the chain‐like H‐bond structures that give rise to the GM. Imidazole also functions well as a parent compound in DES mixtures [38, 39], making it a natural choice for inclusion in a model system.

Conversely, levulinic acid (LA, C_5_H_8_O_3_) is an amphoteric keto acid, notable for its strong hydrogen bonding capabilities [40] and its successful application in DES formulations [39, 41, 42]. Despite its low ionic conductivity and crystallization near room temperature in its pure state due to the carboxylic acid groups' tendency to dimerize, LA's strong hydrogen bonding capabilities make it an ideal complement for imidazole in our model CoHBE [24, 27]. For clarity, we will refer to the resulting DES/CoHBE as functions of increasing the concentration of imidazole in the system, such that all compositions are described on the basis of the molecular percentage of imidazole (“ mol% Im”), with the balance being levulinic acid.

Transport and dynamics in the resulting DES/CoHBE systems were characterized by measuring the physicochemical properties at both macroscopic and microscopic levels. Bulk properties give a collective understanding of all contributing mechanisms within the system, which have been summarized using viscosity (η, from rheology), density (ρ), and conductivity ($\sigma_{0}$, from broadband dielectric spectroscopy (BDS)) to compare the transport and dynamics at different compositions (Figure 2). To understand the local environments and intermolecular interactions responsible for both Grotthuss and vehicular proton diffusion, experimental results were collected for the characteristic relaxation timescales from BDS, diffusion rates & chemical shifts from nuclear magnetic resonance (NMR), and solvent probe reorientation timescales from time‐resolved fluorescence spectroscopy. Finally, small‐angle neutron scattering (SANS) studies on a representative composition (60 mol% Im) at T = 298 K were conducted to obtain insights into the local structural environments.

**FIGURE 1:**
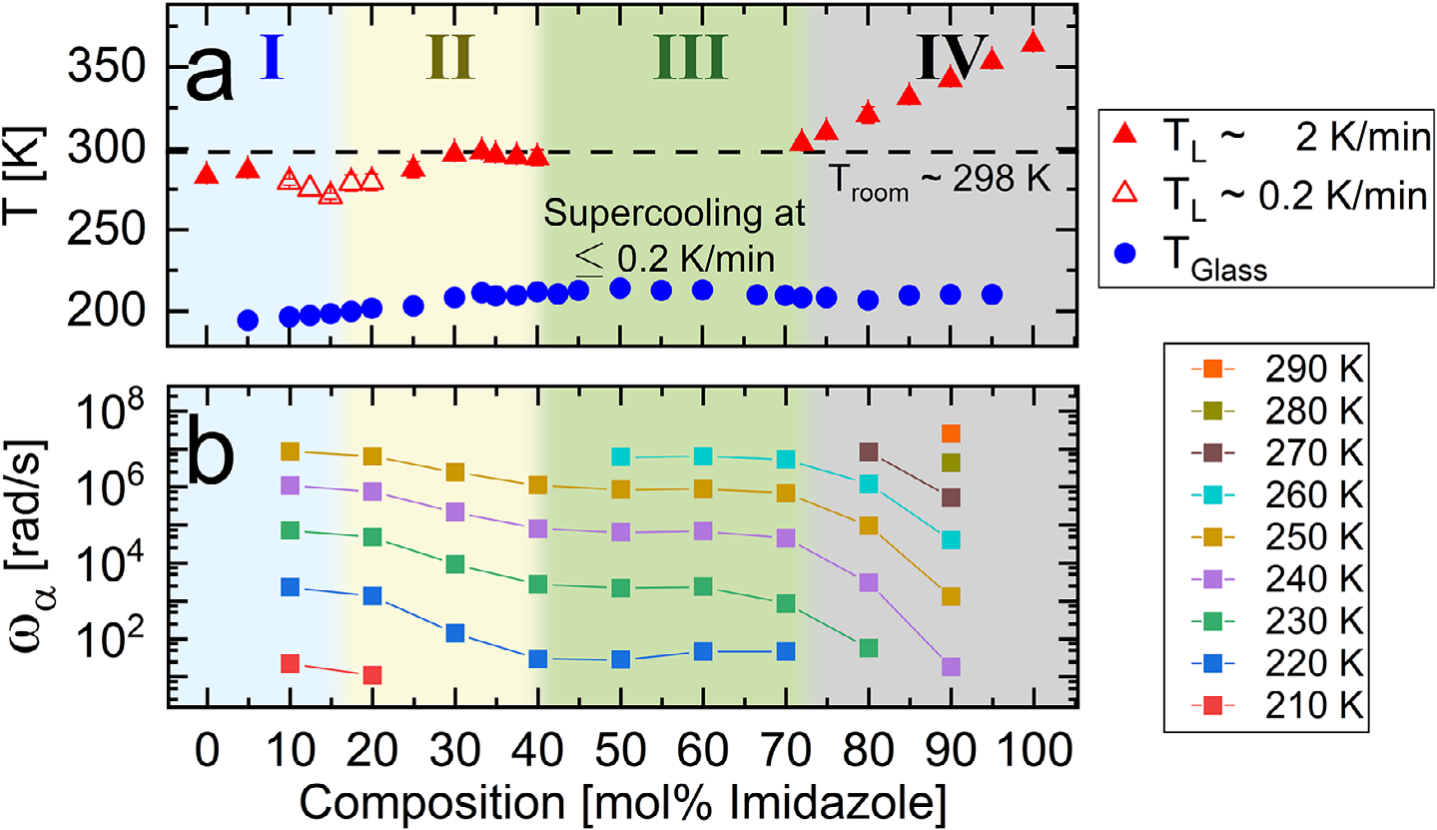
(a) Phase behavior of [Im][LA] DES/CoHBE system, compiled from 0.02–2 K/min temperature‐modulated heating ramps (MDSC) from calorimetry, showing liquidus (*T*_L_) and glass transition temperatures (*T*_g_). Region IV Compositions are not liquid at 298 K and are omitted from further discussion herein. (b) The mean primary structural relaxation rates($\omega_{\alpha }$), obtained from the dielectric loss ($\varepsilon^{\prime \prime }$) peaks from broadband dielectric spectroscopy. Data at multiple temperatures are presented to show that the composition‐dependent trends at lower temperatures can potentially be extrapolated to room temperature (Section 4.3 and Figure S1).

**FIGURE 2:**
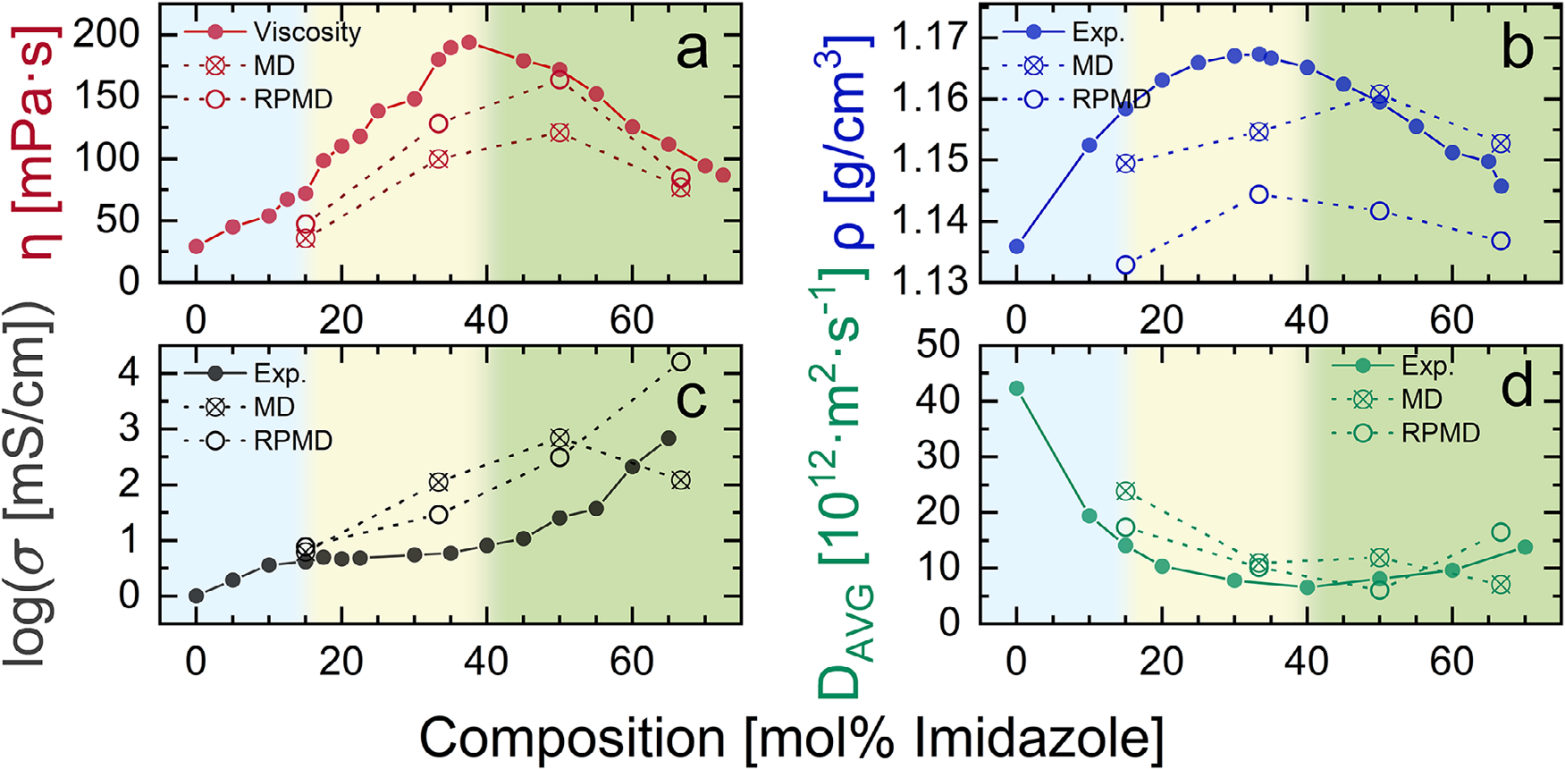
Key bulk transport properties in the [Im][LA] mixture as a function of composition at room temperature [298 K]. Data is given for experiments (filled circles), molecular dynamics (crossed circles), and RPMD simulations (hollow circles), from region I through region III, for (a) viscosity (η), (b) density (ρ), (c) conductivity (σ), (d) and average molecular diffusion coefficients ($D_{AVG}$).

Furthermore, machine learning‐based molecular dynamics (ML‐MD) simulations, including path integral molecular dynamics (PIMD) [43, 44] and ring polymer molecular dynamics (RPMD) [45] simulations, were conducted to account for nuclear quantum effects (NQEs), validate extended experimental observations, and develop a comprehensive understanding of the structure‐property relationships. These advanced simulations were enabled by representing the ab initio potential energy surface with an equivariant transformer machine learning (ML) model [46], trained on density functional theory (DFT) energies and forces from a curated dataset (Section 4.10.2). The computational efficiency of this ML model enabled us to perform expensive path integral simulations with converged statistics.

## 2 Results and Discussion

### 2.1 Initial Characterization

Differential Scanning Calorimetry (DSC) experiments characterized the eutectic phase behavior of the [Im][LA] CoHBE, identifying compositions which are liquid at room temperature (Figure 1a). A local eutectic composition was found around 15 mol% Im, and a region between 40 and 70 mol% Im showed strong supercooling tendencies. For the purpose of discussing the composition‐dependent evolution of the system, we divide the composition space into four behavioral regimes based on experimentally observed crossover points in the liquidus temperatures and conductivity trends. These regimes do not represent distinct thermodynamic phases or topological regions of the material, but rather composition ranges exhibiting qualitatively different transport and phase behaviors. Region I (0–15 mol% Im) showing a local eutectic melting point depression; region II (15–40 mol% Im) with increasing liquidus temperatures; region III (40–70 mol% Im) exhibiting strong supercooling and glass‐forming behavior; and region IV. This final region is primarily solid at room temperature and is excluded from further discussion. Subsequent experiments aimed to contextualize these phase behaviors with bulk transport and molecular dynamics.

### 2.2 Impact of Composition on Bulk Transport Properties

The boundaries between regions I and IV should be interpreted as experimentally identified crossover compositions rather than sharp phase boundaries. As the imidazole concentration increases from 0 to 40 mol% Im (regions I and II), the system shows increasing viscosity (η) and density (ρ), concurrent with decreasing average molecular diffusion rates (Figure 2a–d). These observations collectively indicate a strengthening of the HBN within the [Im][LA] mixture, confirmed by a slowdown in $\omega_{\alpha }$ (Figure 1b) and increasing chemical shift of the exchangeable proton (Figure 3b), due to increase imidazole‐levulinic acid interactions. While conductivity sharply increases in region I due to increased number density of charge carriers, its rise slows and eventually plateaus in region II, attributed to competition between rising charged species concentration and increasing viscosity, which impedes vehicular transport. Region III shows another sharp rise in the conductivity as the imidazole concentration increases, which gives rise to the formation of extended hydrogen‐bonded chains, as discussed in Section S1.5. The role of these chains in acting as dynamical conduits for proton transport and time scales for chain reorientation are also described in Section S1.5.

**FIGURE 3:**
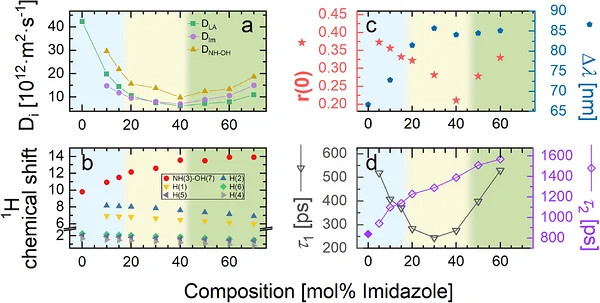
Composition dependence of local transport properties at T = 298 K. (a) Vehicular diffusion constants obtained from PFG‐NMR for levulinic acid ($D_{LA,exp}$), imidazole ($D_{Im,exp}$), and acidic hydrogen atoms ($D_{NH-OH,exp}$). $D_{Im,exp}$ and $D_{LA,exp}$ approach and crossover in Region II. Average diffusion rates for the acidic protons $D_{NH-OH,exp}$ are faster everywhere. (b) ^1^H NMR chemical shifts. While up‐field shifts of most labeled protons (1,2,4,5,6) indicate trends from composition‐weighted contributions [47], the (3)(7) peak corresponds to the indistinguishable proton shared between [Im][LA] via NH⋯OH interactions. The downfield shift of this peak across Regions I, II indicates the development of a strong HBN between the constituent species, which remains strongly de‐shielded across Region III. (c) *Left* Fluorescence anisotropy measurements (r(0)) give a measure of the level of interaction of the *local* environment with the Nile Red (NR) probe. The initial value at 0 mol% Im is 0.149, meaning the introduction of *any* imidazole immediately results in a far more restricted NR probe. *Right* The Stokes shift serves as an indicator of the confinement and/or structuring around NR. (d) Fast ($\tau_{1}$, hollow gray inverted triangles) and slow ($\tau_{2}$, hollow purple diamonds) solvent relaxation components obtained from time‐resolved fluorescence measurements using a Nile Red (NR) probe.

NMR diffusion measurements show that the apparent diffusion coefficient of the exchangeable NH–OH hydrogen, $D_{\mathrm{NH}-\mathrm{OH}}$, is faster than the molecular diffusion of imidazole and levulinic acid across all compositions. This enhanced apparent mobility is attributed to proton relocation through exchange/reactive hopping between hydrogen‐bonding sites, superimposed on the vehicular diffusion of the molecular species. However, a direct quantitative contribution ratio of NH–OH exchange to the overall diffusion process cannot be extracted unambiguously from the present PFG NMR data alone. Therefore, $D_{\mathrm{NH}-\mathrm{OH}}$ should not be interpreted as the vehicular diffusion coefficient of a distinct molecular species, but rather as an apparent diffusion coefficient that reflects both molecular diffusion and exchange‐mediated proton relocation.

### 2.3 Impact of Composition on Local Transport Properties

In region III, the relationship between viscosity/density and conductivity/diffusion appears conventional (Figure 2), but the thermodynamic phase data present a paradox. The existence of a glass‐forming liquid phase with negligible propensity to crystallize above the glass transition throughoutrRegion III (Section 4.2 and Figure 1a) suggests a change in the local packing presumably due to the formation of long‐lived HBN structures. Measures of thermodynamic and local transport properties, including plateaus in Tg, $\omega_{\alpha }$ (Figure 1a,b), and 1H chemical shift, Stokes shift (Figure 3b,c), confirm strong, fully developed HBNs above 40 mol% Im. Experimental measures of local dynamics show the system largely unchanging or becoming less mobile across region III, while bulk transport probes indicate increasing mobilities.

The paradox of region III bulk transport behavior lies in the contradictions based on local transport trends (molecular dynamics and hydrogen bonding), demonstrating a decoupling between bulk and molecular physicochemical transport. From classical theories, the experimentally observed changes in region III should be possible if vehicular transport is the sole mechanism. However, our Nernst‐Einstein conductivities, calculated using the experimental vehicular diffusion of reactive protons ($D_{NH-OH,exp}$), significantly under‐predict region III conductivity (Figure S11), thus indicating that vehicular diffusion of the proton defects cannot alone account for the observed conductivity trends. On the other hand, RPMD estimates for the excess acidic proton diffusion ($D_{H^{+}}$), accounting for Grotthuss diffusion in addition to vehicular diffusion, coincide with a sharp conductivity rise similar to measured trends (Figure S11).

### 2.4 Evidence of Hydrogen‐Bonding Structure Formation

#### 2.4.1 Confirmation of Chain Structures

Combined with the insights about increased solvent rigidity, “preserved” HBN strengths, and pronounced contributions from GM, structures corresponding to hydrogen bonding chains should be detectable at appropriate length scales. To probe for such structures, we performed small‐angle neutron scattering (SANS) measurements on a representative mixture of deuterated imidazole and levulinic acid in the strongly supercooling region III. The neutron scattering curve of the 60 mol% Im composition at T = 298 K shows a peak at at q ≈ 0.45 Å^−1^, which indicates that a persistent structure emerges in the liquid with an average size of q≈ 13 Å  (Figure 4d). As such, if this feature does indeed originate from the presence of hydrogen‐bonded chains composed primarily of imidazole (which possesses a diameter of roughly ∼3.5 Å), it would correspond to chains that are ∼ 3 – 4 units long on average but can tend toward longer chains as well. These data can be checked against hydrogen bond chain length distribution calculations, where the results of Figure 4c indicate average chain lengths of ∼4 units in region III, which agree with the estimate of chain lengths obtained from SANS. Details of the hydrogen‐bond chain length distribution calculations from distance and angle free energy surfaces are provided in Section S1.8.

**FIGURE 4:**
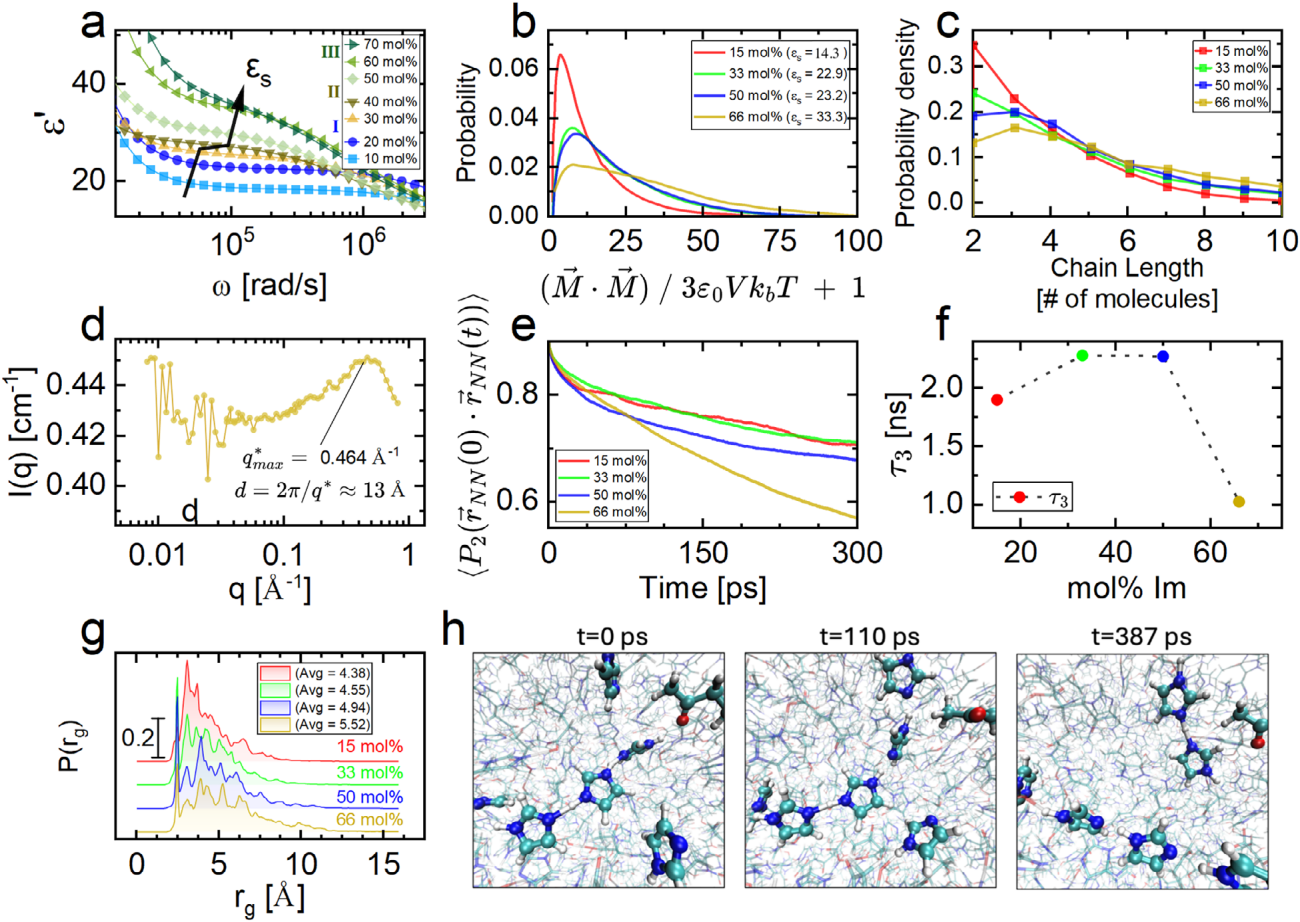
Experimental and simulations results covering aspects dictating the formation + behaviors of HBN chains. (a) Representative trends in static dielectric permittivity ($\varepsilon_{s}$) from BDS. $\varepsilon_{s}$, which are a measure of the polarizability of the material, are obtained from a plateau in the real permittivity signal ($\varepsilon^{\prime }$) (Experiments were performed at 250 K to avoid electrode polarization at higher temperatures). (b) Composition‐dependent trend in $\varepsilon_{s}$ obtained from PIMD simulations match the experimental trend in *a)*. (c) Distribution of hydrogen‐bonded chain lengths obtained from PIMD simulations. The average chain lengths follow the same trend as the simulated and experimental $\varepsilon_{s}$ from *a & b)*. The incorporation of NQEs also gives longer chains at all compositions (Table S3). (d) Small angle neutron scattering of the 60 mol% Im d‐Im/LA mixture at 298 K. Gaussian fitting of a clear peak at $q^{*}∼$ 0.464 Å^−1^ gives a structure on the order of 13 Å. (e, f) The orientational correlation function of an imidazole molecule's ${\vec{r}}_{NN}$ vector over time, indicating how its orientation at time t correlates with its initial orientation. Even though 66 mol% Im presents the longest hydrogen‐bonded chains, the individual imidazole molecules that make up these chains reorient relatively quickly, as evidenced by the lower extracted decay timescale $\tau_{3}$ at 66 mol% Im. (g) Probability distribution of the radius of gyration of hydrogen‐bonded chains in different imidazole concentrations. (h) Representative snapshots obtained from the centroid trajectory of our RPMD simulations demonstrating chain rearrangements involving individual imidazole molecules while the rest of network remains relatively intact.

### 2.5 Crucial Insights From MD Simulations

Discrepancies between MD and PIMD simulations make these results especially significant. Proton transfer free energy barriers (Figure S3a,b) highlight the critical role of Nuclear Quantum Effects (NQEs) in determining system properties. When NQEs are neglected, proton transfer free energy barriers are considerably overestimated, with the imidazole‐imidazole barrier (Figure S3b middle) around ∼1.5 $k_{B}T$. PIMD simulations, accounting for NQEs, show lower imidazole‐imidazole barriers at ∼0.5 $k_{B}T$, rendering them more thermally accessible and conducive for synchronized reactive proton hops characteristic of a Grotthuss mechanism.

To enable appreciable acceleration of charge transport via a Grotthuss mechanism, hydrogen‐bonded chains must be structurally stable enough to support extended structures, yet flexible enough to allow rearrangements that cut off backward diffusion of charges. While experimental timescales from glass transition temperatures, time‐resolved fluorescence, and BDS experiments Figures 1, 2, 3 show slower collective re‐orientations with increasing mol% Im, consistent with longer chain formation, this appears at odds with the decreasing viscosity and increasing diffusivities observed in region III Figure 2. Our RPMD simulations provide crucial insights, showing that the fitted decay time to the orientational time correlation function of imidazole molecules roughly follows the trend in viscosities (Figure 4e,f vs. Figure 2a). These dynamical results indicate that the extended hydrogen‐bonded chains in Region III are structurally stable and persistent at long timescales, but simultaneously quite fluid with respect to their constituent molecules. This mechanistic picture is consistent with the trends in dipole moments ($\varepsilon_{s}$) which become longer than individual molecules through cooperative alignments (Figure 4a), and with the slowing timescales of collective chain rearrangements with increasing mol% Im in region III. This behavior was captured in Figure 4h, where snapshots from our RPMD simulation for 66 mol% Im demonstrating the flipping of an imidazole molecule from one chain to another. In other words, because individual imidazole molecules can reorient faster than the collective chain dipoles, no individual “snapshot” of associated links forming a chain persists for long, but extended chains persist overall thanks to the role of imidazole serving as “railroad track” switches between many possible pathways, effectively promoting forward Grotthuss diffusion of protons.

## 3 Conclusions

Our investigation of the [Im][LA] system demonstrates a clear decoupling between bulk and local transport properties, highlighting the pivotal role of emergent hydrogen bonding chains in this CoHBE as key to promotingthe Grotthuss proton transport mechanism. We identify three distinct composition ranges, each exhibiting unique transport behaviors:

- **Region I (0–15 mol% Im)**: Conductivity initially increases sharply due to the introduction of charge carriers, despite slowing vehicular diffusion.
- **Region II (15–40 mol% Im)**: The system approaches maximum packing and viscosity, causing vehicular diffusion's contribution to saturate. The continued (sluggish) monotonic increase in conductivity, then, is due to the increasing emergence of Grotthuss diffusion via extended hydrogen‐bonded chains, which somewhat offsets the congestion from viscosity and density in Region II.
- **Region III (40–70 mol% Im)**: This region shows a rapid increase in conductivities, predominantly driven by enhanced Grotthuss diffusion of protons. This acceleration is attributed to both increasing hydrogen‐bonded chain length and the faster reorientation timescales of individual imidazole molecules within these chains.

The behaviors in Region III are particularly interesting, and present an apparent paradox: despite slower *collective* reorientational timescales of the system and the overall dipole moment increases (Figures 1b and 4a), *individual* imidazole molecules within these structural segments can reorient and flip between chains on a much faster timescale (Figure 4e,f and Table S2). This dynamic behavior, which acts like “railroad track” switches, actively promoting the forward Grotthuss diffusion of protons. The tendency of imidazole to form chains within a persistent, fully developed HBN results in favorable changes in packing, which causes the decrease in densities, viscosities, and increasing diffusion rates in Region III. As such, the faster reorientation and preference of imidazole for chain formation result in greater bulk mobilities in addition to the conductivity increases from the emergent GM. The persistence of a fully‐developed network in conjunction with the formation of said chains explains both the paradoxical reduction in viscosity through Region III, *and* the strong supercooling tendency of Region III; in other words, the strong HBN + extended chain formation + fast individual molecular re‐orientations also provides access to far more liquid compositions, over much wider temperature windows, than would be possible without (given that the “true” eutectic point before the onset of H‐bonding chains lies closer to 15 mol% Im).

Our machine‐learning driven molecular dynamics simulations, particularly those incorporating NQEs via PIMD, were crucial for validating these experimental conclusions (Figure 2). Simulations demonstrate that the reactive proton diffusion coefficients are enhanced by the GM across all three regions, but NQEs in particular largely promote the protonation of imidazole species. They consequently both catalyze chain formation and influence hydrogen‐bond chain reorientation, all of which drive Grotthuss diffusion, particularly in region III. As such, our analysis confirms that the monotonic increase in proton diffusion coefficients seen experimentally correlates with the increasing average length of the hydrogen‐bonded chains in these electrolyte systems (Figure 4). Critically, classical MD simulations (omitting NQEs) predicted considerably higher proton transfer barrier, longer hydrogen bonds (see Figure S3), and shorter hydrogen‐bonded chains than were observed either experimentally or via the PIMD simulations, underscoring the importance of not neglecting NQEs in simulations of future CoHBEs.

In conclusion, our combined experimental and simulation efforts, particularly simulations incorporating NQEs, have successfully disentangled the distinct microscopic factors contributing to the composition‐dependent conductivity trends observed in [Im][LA]. Consistent with prior studies of similar strongly hydrogen‐bonded liquids [15, 18, 19, 27, 28, 37, 48], we affirm that the rapid reorientation of individual molecules within chains in a collectively slow‐reorienting HBN is responsible for the improvements in both transport and bulk dynamics across the [Im][LA] composition space. Our results show that several factors contribute to the observed phenomena: thermally accessible proton transfer barriers, the formation of extended hydrogen‐bond chains, a strong preference for imidazole protonation, dynamic, nanosecond‐timescale rearrangement of individual molecules, and nuclear quantum effects (NQEs). Together, these not only produce GM‐dominant conductivity improvements, but also stabilize the liquid state of region III and enhance its bulk transport properties. The results and their conclusions presented here apply beyond the present context to broader classes of concentrated hydrogen‐bonded systems composed of molecular species capable of accepting and donating single hydrogen bonds and transporting protons via chain‐like structures [14, 15, 18, 19, 27, 28] and accords with these previous studies, which revealed the importance of chain formation and reorientation, while pointing toward new mechanistic features. The present study constitutes an example of the type of synergistic investigation involving a range of experimental techniques and quantum simulation capable of elucidating and validating the underlying structure, dynamics, mechanisms, and decoupling of charge transport kinetics from solvent dynamics that drive the Grotthuss mechanism in hydrogen bond networks.

## 4 Methods

### 4.1 Sample Preparation

Anhydrous imidazole (99%) and pure levulinic acid (98%) were obtained from Sigma Aldrich. Constituents were dried in a vacuum oven for 24+ h at 323 K. Samples were made and handled between experiments in a nitrogen‐purged glove box. Compositions using these constituents were made on a 1 mole basis, reported herein as “mol% Im,” with errors ≤±0.5%. Samples were sealed and heated on a hot plate with stir bars at ≥ 360 K until fully melted and homogeneous. The water content of resulting liquid compositions was vetted with a HI903 Karl Fischer Volumetric Titrator to ensure that only samples with water contents of ≤ 2 mg/g were used in further testing.

Compositions were initially made in 10 mol% Im increments, with additional compositions prepared as needed to increase resolution in composition spaces of interest. For NMR, ACS reagent grade ^1^H‐imidazole (≥ 99%, Fisher Scientific Co.) and levulinic acid (≥ 98%, Thermo Scientific Chemicals) were used. NMR reagents were dried for ≥ 5 days at 333 K and stored within an inert argon atmosphere before use.

For time‐resolved fluorescence, a small quantity of Nile red (NR) dye was introduced into each composition to achieve an optical density of 0.5 over a 0.2 cm path length.

### 4.2 Differential Scanning Calorimetry

DSC experiments were performed using a TA Instruments DSC2500 unit with the RCS 120 cooling accessory. Samples were hermetically sealed in aluminum TZero calorimetry pans under nitrogen. Temperature modulated differential scanning calorimetry experiments (MDSC) were performed at 2 K/min ramp rates over ranges between 150 to 400 K for each sample, with replicates. Glass transition temperatures (*T*_g_) were identified at the midpoint of the transition in the reversing heat capacity from MDSC, accounting for thermal history dependence and enthalpic relaxation [49, 50]. Liquidus temperatures (*T*_L_) were defined as the endset of the last discernible melt event, as “melting temperatures” (*T*_m_) are insufficient descriptors of eutectic systems with dual‐phase regions [51]. Replicate experiments were performed using conventional heat‐cool‐heat (HCH) DSC cycles as an additional check of repeatability and absence of hystereses.

Portions of region I and all of region III readily supercooled at 2 K/min rates, yielding no freeze/melt transitions. In these regions, tests at slower rates were used in an attempt to avoid supercooling, with error bars scaled to permit comparison against 2 K/min results (indicated accordingly in Figure 1a). The local eutectic point at 15 mol% Im in region I was determined with 0.2 K/min cooling rates, but region III still would not freeze. A final test run for several days on the 60 mol% Im composition at 0.02 K/min rates with replicates still showed no freezing, confirming the supercooling tendency of region III. Errors are within the symbol sizes unless otherwise indicated.

### 4.3 Broadband Dielectric Spectroscopy

BDS measurements were performed using a Novocontrol Alpha‐A High Performance Frequency Analyzer with a Quatro temperature control system. Measurements were taken in a frequency range spanning 10^−1^ to 10^7^ Hz. Parallel steel electrodes were separated with 0.3 mm thick Teflon spacers. Samples were loaded between electrodes without air pockets. The Quatro temperature control system maintained temperature stability of ± 0.1 K during isothermal holds while frequency sweep measurements were taken.

Characteristic relaxation values were obtained from the complex permittivity, $\varepsilon^{*}\left(\omega \right)=\varepsilon^{\prime }\left(\omega \right)-i\varepsilon^{\prime \prime }\left(\omega \right)$, while the dc ionic conductivity of the material was determined from the frequency‐independent regime from the real portion of the complex conductivity, $\sigma^{*}\left(\omega \right)=\sigma^{\prime }\left(\omega \right)+i\sigma^{\prime \prime }\left(\omega \right)$. These functions are related by $\varepsilon^{*}\left(\omega \right)=i\omega \varepsilon_{0}\sigma^{*}\left(\omega \right)$, where $\varepsilon_{0}$ is the permittivity of free space. Dielectric relaxations are often overshadowed by dc ionic conductivity contributions at low frequencies due to electrode polarization. Therefore, it is common to turn to the derivative representation of the dielectric loss, 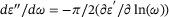, to uncover the underlying spectral shapes [50]. $\omega_{\alpha }$ values are extracted as the peak position of fits of these plots (Figure S1).

### 4.4 Frequency Flow Sweep Rheology

Room‐temperature viscosities were determined for each liquid composition as the value in the Newtonian region, averaged over shear rates from 10^−1^ to 10^3^ $s^{-1}$ with an accuracy of ±3 mPa·s using a TA Instruments ARES‐G2 rheometer fitted with 25 mm parallel plates at 298 ± 0.1 K. Temperature control was achieved with the forced convection oven (FCO) accessory.

### 4.5 Pycnometry Measurements

Liquid sample densities were measured at 298.15 K (± 0.02 K) using a vibrating U‐tube density meter (Anton Paar, DMA 4500 M) with an uncertainty of ± 0.00005 g/cm^3^. Density of solid ^1^H‐imidazole was measured with a pycnometer (Borosilicate 3.3 Glass ‐ Eisco Labs; 10 ± 0.1 mL) from 378.15 – 393.15 K (± 0.1 K) in 5 K increments (Isotemp Oven 282A). Masses were weighed using a Sartorius Quintix313‐1S Analytical Balance (±0.001 g). Densities were determined by $\rho =(m_{f}-m_{g})/V$, where $m_{f}$ is the filled pycnometer mass, $m_{g}$ is the empty pycnometer mass, and V is the pycnometer volume. Melted samples were allowed to overflow for complete filling, and residual overflow was removed. A 1 h thermal equalization time was observed between measurements. Propagation of error analysis, considering uncertainties in pycnometer volume, temperature, and weighted masses, yielded an uncertainty of ± 0.01 g/cm^3^ for ^1^H‐imidazole density.

### 4.6 Nuclear Magnetic Resonance Characterization

Samples were placed in 5 mm NMR tubes and flame‐sealed. ^1^H NMR and Pulsed Field Gradient NMR (PFG‐NMR) experiments were conducted on a Bruker Avance 400 NMR console (9.4 T) with a 5 mm pulsed‐field z‐gradient BBFO iProbe. For each sample, the probe was carefully tuned, and the signal following 90°pulses was evaluated. Sample temperature was controlled using a variable temperature control unit via air gas flow. Standard Bruker library programs were used.

The ^1^H spectra were externally referenced to a 1% CHCl_3_ in acetone‐d_6_ standard (8.02 ppm). PFG‐NMR measurements were carried out at 298 K in the ^1^H domain using the bipolar pulse longitudinal eddy current delay (BPP‐LED) pulse sequence. All experiments used 8 transients per increment with 16384 points in the F_2_ dimension, over a spectral width of 24 ppm. Relaxation delay was set to at least five times T_1_, with 8 dummy scans. Pulse gradients were incremented from 2% to 95% of the maximum strength (50 G/cm) in 16 steps. Magnetic field pulse gradient duration (δ, 5–6 ms) and diffusion times (Δ, 0.2–0.8 s) were optimized for each experiment to achieve 95% signal attenuation for the slowest species. An additional experiment for pure imidazole was performed at 385 K (δ=1.5 ms, Δ=0.1 s). Raw spectra were manually phased and baseline corrected. Data were processed using an exponential filter in F_2_ dimension (LB = 0.3 Hz). Integrals were employed in the Bruker T_1_/T_2_ module of TopSpin to fit the Stejskal–Tanner relation [52]:

$$I=I_{0}\cdot \exp\left(-D\;\gamma^{2}\;g^{2}\;\delta^{2}\left(\Delta \tau -\frac{\delta }{3}\right)\right)$$

where I is the signal intensity with the gradient applied, $I_{0}$ is the initial signal intensity, γ is the gyromagnetic ratio of the investigated nucleus, g is the gradient strength applied, D is the diffusion coefficient obtainable from integral fits of the NMR spectra, and Δτ is the delay between the pulses.

### 4.7 Steady‐State Absorbance and Emission (Stokes Shift Measurements)

Steady‐state absorption spectra were recorded using a Varian Cary 50 UV‐Vis spectrophotometer, and steady‐state fluorescence measurements with a Varian Cary Eclipse fluorescence spectrophotometer. The excitation wavelength was set to 510 nm. Slit widths for excitation and emission were adjusted to 2.5 and 5 nm, respectively, and the photomultiplier tube voltage was set to 600 V. All experiments were conducted at room temperature (298 K) and ambient pressure.

### 4.8 Time‐Resolved Fluorescence

Time‐resolved fluorescence was measured using an Optronis SC‐10 streak camera, triggered by a laser pulse for picosecond resolution over nanosecond windows. The 510 nm excitation wavelength was generated using a TOPAS‐Prim/NirUVis module (Light Conversion), pumped by a ClarkMXR CPA‐2010 laser (780 nm fundamental, 130 fs pulse duration, 1 kHz repetition rate, 850 mW output power). Samples were placed in 2 mm cuvettes and maintained at room temperature (298 K) and ambient pressure. After photoexcitation, the emitted light was directed through a Spectral Products DK240 broadband spectrograph. Camera settings were managed using OptoAnalyse software by Optronis. Data produced an image representing the temporal evolution of fluorescence intensity. Lifetimes were extracted by selecting a narrow region of the image that captured the entire decay, which was then fitted with a bi‐exponential function:

(1) $$A_{1}exp\left(\frac{-t}{\tau_{1}}\right)+A_{2}exp\left(\frac{-t}{\tau_{2}}\right)+y_{0}$$

The amplitudes ($A_{1},A_{2}$), time constants ($\tau_{1},\tau_{2}$), and baseline shift ($y_{0}$) are fitting parameters. R^2^ values of > 0.99 were obtained from fits of all samples. Fluorescence anisotropy was determined by measuring initial anisotropy value, r(0), with polarizers placed before and after the sample.

### 4.9 Small Angle Neutron Scattering

Small‐angle neutron scattering (SANS) measurements were conducted at room temperature using the NGB 30‐meter SANS instrument at the High Flux Isotope Reactor (HFIR) at Oak Ridge National Lab (ORNL). Samples of deuterated imidazole and protonated levulinic acid were prepared prior to arrival, sealed in airtight vials, and subsequently transferred in a glove box to titanium compression cells (1 mm path length) with an O‐ring seal provided by ORNL. An average neutron wavelength of 4.75 Å was employed. The isotropic two‐dimensional (2D) scattering data were converted into one‐dimensional (1D) scattering intensity as a function of the wavevector, q=4πsin(θ/2)/λ, where λ is the neutron wavelength and θ is the scattering angle. The acquired scattering data were corrected for empty cell scattering, detector nonlinearity, electronic noise, stray scattering, sample transmission, and incoherent background scattering. Subsequently, the data were normalized to absolute differential scattering cross‐section and reported in cm^−1^.

### 4.10 Molecular Dynamics Simulations

#### 4.10.1 Ab Initio Molecular Dynamics Simulations

AIMD simulations were conducted for benchmarking and initial ML model training. Three mixtures (33, 50, and 66 mol% Im Im) were prepared. Initial structures were generated using Packmol [53] with 20:40, 30:30, and 40:20 imidazole:levulinic acid molecules. CP2K v 7.1 [54] was used for geometry and simulation cell optimizations (1 bar external pressure), yielding final periodic structures with box side lengths of 18.496, 20.000, and 20.500 Å, respectively. AIMD simulations were performed at 300 K and 383 K using CP2K, with equilibration and subsequent sampling of the NVT ensemble conducted with a massive Nosé–Hoover chain [55] thermostat of length 6. For each mixture and temperature, ∼45 ps of AIMD simulations were run.

For each periodic system, electronic ground state energies, atomic forces, and stress tensors were calculated using density functional theory (DFT) with the PBE exchange‐correlation functional [56] and Grimme D3 [57] dispersion corrections. The mixed Gaussian and plane wave method [58, 59] was employed using the molecularly optimized triple‐ζ valence (TZVP) [60] basis set and an auxiliary plane wave basis cutoff of 450 Ry. Goedecker‐Teter‐Hutter (GTH) pseudopotentials [61] were used to reduce computational costs.

#### 4.10.2 Machine Learning Model for Potential Energy Surfaces

An equivariant transformer (ET) neural network [62] was trained to DFT energies and forces to serve as a more computationally efficient proxy for the latter. Each chemically distinct atom was assigned its own atom type with the reactive proton, whether instantaneously bonded to imidazole or levulinic acid, being its own atom type. For inference, the information content of these atomic embeddings are refined with 2 update layers that employ a modified attention mechanism consisting of 4 attention heads [62]. In all, the ET model consists of 171 thousand trainable parameters. Training was conducted using the TorchMD‐Net package, v2.00 [63].

Query‐by‐committee (QbC), an active learning procedure commonly used to develop ML models for condensed phase systems [64, 65], was employed to iteratively and judiciously construct the dataset for training our ET model. The procedure started with training and validation sets each consisting of 150 random configurations sampled from the three 383 K AIMD simulations described in Section 4.10.1 (see Table S1). A committee of 4 ET models, each with a different random initialization of their trainable parameters, was trained to this dataset while employing early stopping in order to mitigate overfitting. The resulting ET model with the lowest validation set loss was chosen to run short MD simulations (ML‐MD) for the 33, 50, and 66 mol% Im systems. These short ML‐MD simulations were run at 383 K for up to 25 ps but stopped earlier if the simulations became unstable (e.g., chemically unlikely bond breaking events). Aside from the differences in temperature and duration, these ML‐MD simulations employed the same settings as used for the equilibration runs described in Section 4.10.3. The MD‐sampled configurations with the largest disagreement (i.e., standard deviation) in potential energy predictions among the committee of models were added to the training set for the next iteration of the QbC procedure. At most 10 configurations per system were added to the training set per iteration, with a 100 fs spacing between selected configurations to avoid adding overly similar configurations. ML‐MD benchmarks using our ET model trained to this dataset reproduce the AIMD radial distribution functions and proton transfer free energy barriers (see Figure S4). We subsequently continued this procedure but instead employing ML‐PIMD simulations (see Section 4.10.3 for details) run for up to 12.5 ps instead of ML‐MD simulations to sample additional configurations to add to our dataset. We also added configurations of 15 mol% Im and of gas‐phase imidazole, levulinic acid, and possible dimers of the two. A breakdown of the dataset used to train and validate our ML potentials is provided in Table). The RMSE of the predicted energies and atomic forces for the validation set were 2.47 meV/atom and 58 meV/Å, respectively. Correlation plots showing the accuracy of the predicted energies and forces with respect to the DFT reference values are provided in Figure S5.

#### 4.10.3 ML‐MD and ML‐PIMD Simulations

MD simulations employing our ML potential energy surfaces were conducted for systems with 15, 33, 50, and 66 mol% Im with the corresponding experimental densities as reported in Figure 2. Each periodic simulation box contained 60 total molecules, split accordingly between imidazole and levulinic acid depending on the composition. All MD simulations were run using the i‐PI [66, 67] package interfaced to TorchMD‐Net [62, 63].

Classical ML‐MD simulations in the NVT ensemble for each system (15, 33, 50, and 66 mol% Im) at 298 K were first equilibrated for 50 ps using the global canonical sampling through velocity rescaling (CSVR) thermostat [68] with a time constant of 0.1 ps and a timestep of 0.5 fs. Each of these simulations was then continued for 200 ps with the same CSVR thermostat saving configurations every 10 fs. These sampled configurations were used to compute the ML‐MD static properties we have reported here. Classical ML‐MD simulations in the NVE ensemble for each system were initialized with the last configuration sampled from the aforementioned NVT simulations. The configurations and the instantaneous stress tensors were saved every 2.0 fs.

PIMD and RPMD simulations using our ML potentials (ML‐PIMD and ML‐RPMD) were conducted to investigate the effect of incorporating nuclear quantum effects (NQE). The ML‐PIMD simulations in the NVT ensembles were run using 20 replicas, a 0.25 fs timestep, and the global version of the path integral Langevin equation thermostat (PILE‐G) [69] with a time constant of 0.1 ps for the centroid. The relevant static properties reported in this work for each system were obtained from ML‐PIMD simulations run for 250 ps, saving replica configurations every 10 fs, and preceded by a 25 ps equilibration run. ML‐RPMD simulations in the NVE ensemble for each system were initialized with the replica configurations sampled from the last step of the aforementioned NVT simulations. The configurations of the replicas and the instantaneous stress tensors were saved every 2.0 fs.

To obtain our density estimates, we performed both ML‐MD and ML‐PIMD simulations in the NPT ensemble for each system (15, 33, 50, and 66 mol% Im) at 298 K and 1 bar. In both cases for the atomic degrees of freedom, we used the same thermostat settings as employed when we sampled NVT. For our ML‐MD simulations sampling the NPT ensemble, we employed the Bussi‐Zykova‐Parrinello (BZP) barostat [70] for the simulation cell degree of freedom employing a barostat relaxation time constant of 0.5 ps and cell‐specific white noise Langevin thermostat with a relaxation time constant of 0.5 ps. For our ML‐PIMD simulations we employed the path integral extension of the BZP barostat as implemented in i‐PI [66].

For the calculation of the instantaneous stress tensors required for these simulations, we implemented the calculation of the virial term [71] in our custom version of TorchMD‐Net. The RMSEs on the validation set were 0.12 GPa and 0.023 GPa for the diagonal and off‐diagonal components of the stress tensor. Correlation plots showing the accuracy of the predicted stress tensors with respect to the DFT reference values are provided in Figure S5. For the PIMD stress tensors, we employed the path integral virial estimator [44, 66, 72].

## Conflicts of Interest

The authors declare no conflicts of interest.

## Supporting information

The supporting information(SI) document includes additional computational details, additional plots relating to the hydrogen bond statistics, free energy profiles from the MD and RPMD simulations, and detailed time correlation functions and mean‐square displacement plots. **Supporting File**: advs77781‐sup‐0001‐SuppMat.pdf.

## Data Availability

The data that support the findings of this study are openly available in Open Science Framework at http://osf.io.
