# Supplementary material for: Nonlinear Quantum Effects Drive Grotthuss Proton Conduction in Structured Electrolytes Based on Deep Eutectic Solvents
Source: Adv Sci (Weinh). 2026 Oct 8:e77781. Online ahead of print. doi: 10.1002/advs.77781 (PMC13648571; doi:10.1002/advs.77781)
Supplement: Supplementary file 1 [file ADVS-9999-e77781-s001.pdf]

Supporting Information: Nonlinear Quantum Effects Drive Grotthuss Proton Conduction in Structured Electrolytes Based on Deep Eutectic Solvents

Benworth B. Hansen[1], Michael S. Chen[4,8], Giselle de A. L. e Souza[2], Kaylie Glynn[1], Alan Robledo[4], Emilia Pelegano-Titmuss[2], Katherine Hightower[4], Miguel Muñoz[3], Rathiesh Pandian[3], Clemens Burda[3], Aikaterini Sanida[1], Mark Dadmun[9], Burcu E. Gurkan[3], Steve G. Greenbaum[2], Mark E. Tuckerman[4,5,6,7,8*], Joshua R. Sangoro[1*]

[1*]William G. Lowrie Department of Chemical and Biomolecular Engineering, The Ohio State University, 151 W. Woodruff Ave., Columbus, 43210, OH, USA.

[2]Department of Physics and Astronomy, Hunter College of City University of New York, 695 Park Ave., New York, 10065, NY, USA.

[3]Department of Chemical and Biomolecular Engineering, Case Western Reserve University, 10900 Euclid Ave., Cleveland, 44106, OH, USA.

[4]Department of Chemistry, New York University, 24 Waverly Place, 3rd Floor, New York, 10003, NY, USA.

[5]Department of Physics, New York University, 726 Broadway,New York, 10003,NY,USA.

[6]Courant Institute of Mathematical Sciences,New York University,251 Mercer Street,New York,10012, stateNY, USA.

[7]NYU-ECNU Center for Computational Chemistry at NYU Shanghai, Shanghai, 200062, China.

[8]Simons Center for Computational Physical Chemistry, New York University, New Yori, 10003, NY, USA.

[9]Department of Chemistry, University of Tennessee, 1420 Circle Dr., Knoxville, 37996, TN, USA.

*Corresponding author(s). E-mail(s): mark.tuckerman@nyu.edu;
sangoro.1@osu.edu;
Contributing authors: hansen.663@buckeyemail.osu.edu;
mc10050@nyu.edu; giselle.souza@hunter.cuny.edu;
glynn.64@buckeyemail.osu.edu; ar6138@nyu.edu;
emilia.pelegano-titmuss@macaulay.cuny.edu; kh3397@nyu.edu;
mam618@case.edu; rrp45@case.edu; burda@case.edu; sanida.1@osu.edu;
dad@utk.edu; beg23@case.edu; sgreenba@hunter.cuny.edu;

1 Calculation details for properties obtained from simulations

1.1 Free energy barriers

Free energy barriers for the proton transfer reactions between imidazole and levulinic acid were computed along the reaction coordinate $\delta = r_{NH^*} - r_{OH^*}$, where H^* represents the proton shared in a hydrogen bond between imidazole and levulinic acid while r_{NH^*} and r_{OH^*} represent the distances of that proton to the nearest nitrogen and oxygen atoms. $\delta < 0$ when H^* is covalently bonded to imidazole, and vice-versa. Free energy barriers describing the imidazole-imidazole and levulinic acid-levulinic acid are computed for analogous delta coordinates. The corresponding free energy barriers $F(\delta) = -k_B T \ln(P(\delta))$ are computed from the probability distributions of the delta coordinate, $P(\delta)$, as obtained from the set of configurations sampled with our MD simulations. For our ML-PIMD free energy surfaces, bead configurations were used to construct $P(\delta)$.

1.2 Static dielectric permittivities

Static dielectric permittivities were computed from the ensemble average of the squared magnitude of the systems' total dipole moment $\vec{M}$,

$$\epsilon_s = \frac{1}{3\epsilon_0 V k_B T}\langle \vec{M}^2 \rangle + 1, \tag{1}$$

where ϵ_0 is the permittivity of vacuum, V is the volume of the simulation cell, k_B is Boltzmann's constant, and T is the ensemble temperature. We estimated the total dipole moment for a given configuration as the following sum over all N atoms,

$$\vec{M} = \sum_i^N q_i \vec{r}_i, \tag{2}$$

where q_i is the Hirschfeld-I[1, 2] atomic partial charge and $\vec{r}_i$ is the position of atom i. To obtain estimates for q_i in a computationally efficient manner, we employed the same ML architecture we used to model the DFT potential energy surface to instead predict the Hirschfeld-I partial charges obtained from DFT. The same set of configurations for the different bulk electrolyte compositions were used to train and validate this model, with the difference now being that the model is trained to predict the atomic partial charges and their sum, which is just zero for our overall neutral systems, as opposed to the atomic energy contributions and the total energy. The RMSE on the validation set was 0.0071 a.u. Correlation plots showing the accuracy of the predicted atomic partial charges with respect to the reference DFT values are provided in **Figure** S5.

1.3 Diffusion coefficients

Diffusion coefficients were calculated via a linear fit of the long-time slope from 200 to 300 ps of the respective mean square displacement (MSD), using the unwrapped simulated trajectories, and were corrected for system size effects by taking into account the experimental viscosities[3]. Each computed MSD is shown in **Figure** S7. Molecular diffusion coefficients (D_{Im} and D_{LA}) were determined from MSDs of the molecular centers of masses. The vehicular proton diffusion coefficients D_{NH-OH} were determined from MSDs of all potentially reactive hydrogen atoms in the systems, i.e., those covalently bonded to either the carboxylic acid on a levulinic acid or the nitrogen atoms on an imidazole.

The diffusion coefficients for the acidic defects D_{H^+}, which additionally account for Grotthuss, were computed by first identifying all molecules covalently bonded to an acidic proton (or two) at each saved timestep and then for each of those molecules determining whether in the next timestep that same molecule still holds the same acidic proton(s). If not, we determine what molecule received the transferred acidic proton. In tracking the trajectory of these acidic proton defects (i.e., center of mass of the molecule that currently holds that proton), the total number of which is constant and equal to the number of levulinic acid molecules in our simulations, we can then calculate a corresponding MSD and diffusion coefficient. We additionally make sure the trajectory of these acidic proton defects is unwrapped with respect to the periodic boundary conditions employed.

1.4 Conductivities

Conductivities were computed via the Green-Kubo relation,

$$\sigma = \frac{1}{3k_BT}\int_0^\infty \mathrm{d}t\langle \vec{J}(0)\vec{J}(t)\rangle, \tag{3}$$

where V is the volume, k_B is Boltzmann's constant, T is the temperature, and $\vec{J}(t)$ is the collective current,

$$\vec{J} = \sum_i^N q_i \vec{v}_{cm,i}, \tag{4}$$

which is the sum of the molecular charge-weighted velocities with N being the number of molecules in the system, q_i the charge of the molecules, and $\vec{v}_{cm,i}$ is the center-of-mass velocities of the molecules. We estimate the charge of the molecules as the sum of the partial atomic charges as predicted by the same ML model we used in the context of computing static dielectric permitivitties. Given the slowly decaying oscillations in the current time correlation function, we compute the time correlation functions out to a cutoff time t_c=5 ps and fit the remaining long-time decay to $-bt^{-c}$[4]. The current time correlation functions and their fits are shown in **Figure** S9.

1.5 Densities

Average densities were obtained from the ML-MD and ML-PIMD simulations we performed to sample the NPT ensemble for each system (15, 33, 50, and 66 mol% Im) at 298 K and 1 bar. In both cases for the atomic degrees of freedom, we used the same thermostat settings as employed when we sampled NVT. **Figure** S6 shows how the cumulative average of the densities changes with increasing simulation time and highlights the slowly converging nature of the simulated density estimate. The density estimates we report in **Figure 2** are the average density values after 150 ps for each respective simulation.

1.6 Viscosities

The shear viscosities were computed from our MD and RPMD simulations via the following Green-Kubo relation,

$$\eta = \frac{V}{k_B T} \int_0^\infty \mathrm{d}t \langle \mathbf{P}_{\alpha\beta}(0) \mathbf{P}_{\alpha\beta}(t) \rangle, \tag{5}$$

where V is the volume, k_B is Boltzmann's constant, T is the temperature, and $P_{\alpha\beta}$ is an off-diagonal element of the system's stress tensor, i.e., P_{xy}, P_{xz}, and P_{yz}. In addition to the off-diagonal elements, we also make use of linear combination of diagonal elements in computing the time correlation function[5], i.e., $P_{\alpha\beta} = (P_{xx} - P_{yy})/2$, $(P_{xx} - P_{zz})/2$, or $(P_{yy} - P_{zz})/2$. Given the slow decay of the stress tensor time correlation function for these viscous electrolyte systems, we computed the time correlation functions out to a cutoff time of t_c =200 ps and then fit the long-time decay beyond t_c with a stretched exponential of the form $a\mathrm{e}^{-x^b/\tau}$[6]. **Figure** S8 shows the fits of the long-time decays and the resulting running integrals of the viscosities η employing this approach.

1.7 Orientational time correlation functions

Orientational time correlation functions for imidazole molecules with respect to their N-N axes were calculated as follows,

$$C(t) = \langle P_2 \left(\vec{r}_{NN}(0) \vec{r}_{NN}(t) \right) \rangle, \tag{6}$$

where P_2 is the second order Legendre polynomial and $\vec{r}_{NN}$ is the unit vector connecting a given imidazole molecule's two nitrogen atoms. Triexponential fits of these time correlation functions, of the form $a_1 \mathrm{e}^{-t/\tau_1} + a_2 \mathrm{e}^{-t/\tau_2} + (1 - a_1 - a_2)\mathrm{e}^{-t/\tau_3}$, are shown in **Figure** S10 and show good agreement. Biexponential fits to the same time correlation functions are also shown for comparison. The fitted parameters are reported in **Table** S2

1.8 Hydrogen bonded chains

To calculate hydrogen bonded chain lengths from MD simulations, chains were first defined as a set of of molecules such that each pair of neighboring molecules had a

donor-acceptor length, r, and a donor-H-acceptor angle, θ less than or equal to a cutoff value. The cutoff value was determined based on the minimum and maximum values of r and θ of the free energy surface contour $-k_B T \ln P(r, \theta) = 2k_B T$, where $P(r, \theta)$ is the joint probability distribution, following a similar protocol as in Ref. [7]. Separate contour plots were made for classical and quantum simulations as well as for each concentration leading to separate cutoff values. Only chains with an excess proton were considered in the statistics.

References

[1] Bultinck, P., Van Alsenoy, C., Ayers, P. W. & Carbó-Dorca, R. Critical analysis and extension of the hirshfeld atoms in molecules. *The Journal of Chemical Physics* **126**, 144111 (2007). URL https://doi.org/10.1063/1.2715563.

[2] Vanpoucke, D. E. P., Bultinck, P. & Van Driessche, I. Extending hirshfeld-i to bulk and periodic materials. *Journal of Computational Chemistry* **34**, 405–417 (2013). URL https://onlinelibrary.wiley.com/doi/abs/10.1002/jcc.23088.

[3] Yeh, I. C. & Hummer, G. System-size dependence of diffusion coefficients and viscosities from molecular dynamics simulations with periodic boundary conditions. *The Journal of Physical Chemistry B* **108**, 15873–15879 (2004).

[4] Dommert, F. *et al.* A comparative study of two classical force fields on statics and dynamics of [emim][bf4] investigated via molecular dynamics simulations. *The Journal of Chemical Physics* **129**, 224501 (2008). URL https://doi.org/10.1063/1.3030948.

[5] Tuckerman, M. E. *Statistical Mechanics: Theory and Molecular Simulation, Second Edition* (Oxford University Press, Oxford, 2023).

[6] Zhang, Y., Otani, A. & Maginn, E. J. Reliable viscosity calculation from equilibrium molecular dynamics simulations: A time decomposition method. *Journal of Chemical Theory and Computation* **11**, 3537–3546 (2015). URL https://doi.org/10.1021/acs.jctc.5b00351. PMID: 26574439.

[7] Long, Z., Atsango, A. O., Napoli, J. A., Markland, T. E. & Tuckerman, M. E. Elucidating the proton transport pathways in liquid imidazole with first-principles molecular dynamics. *J. Phys. Chem. Lett.* **11**, 6156–6163 (2020).

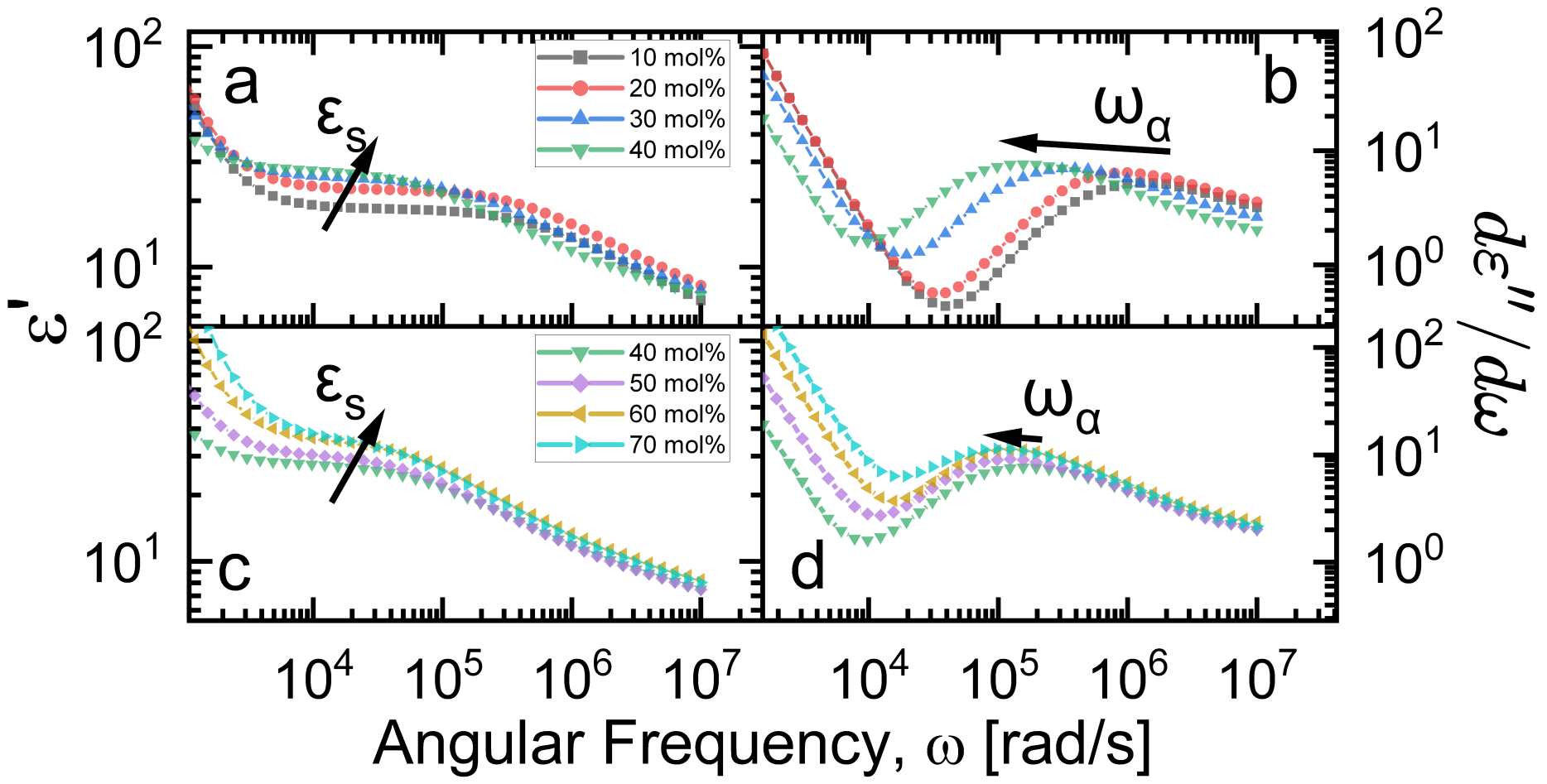

Figure S1 Representative trends in static dielectric permittivity (ε_s) **a,c** and characteristic relaxation rates (ω_α) **b,d** at high and low mol% Im regions from BDS measurements at 250 K. Lower temperature was required for dual purpose of bringing relevant phenomena into experimentally accessible frequency range (see **Section 4.3** on electrode polarization and other parasitic effects), and maintain a supercooled state to avoid cold crystallization of the lowest compositions during the experiment. Real permittivities (ε') can be used to find the static dielectric permittivity (ε_s) of each composition, obtained from the plateau which occurs before the onset of parasitic electrode polarization characteristic of the BDS measurement technique (visible as a transition to a sharp slope on the Increases in ε_s indicate an increase in the polarizability of the material, *i.e.* an increase in the average dipole moment at the molecular scale. The large ε_s increases visible through Region I to Region III indicate dipole moments larger than individual molecules, possible only through vector addition of individual dipoles occurring along linear portions of the hydrogen-bonded network. Critically, a large initial increase in ε_s can be seen through Region I, followed by relatively stagnant growth through Region II before another large increase occurs through Region III, matching conductivity trends in **Figure 2**. ω_α values can be determined from the peak position of ε'.

	15 mol% Im	33 mol% Im	50 mol% Im	66 mol% Im	gas-phase
Train (MD)	20	353	356	311	693
Train (PIMD)	174	40	59	62	0
Validation (MD)	0	0	0	0	77
Validation (PIMD)	10	10	10	10	0

Table S1 Number of configurations for each mol% Im composition, as well as gas-phase configurations, used for the training and validation sets for developing our ML model potential energy surface

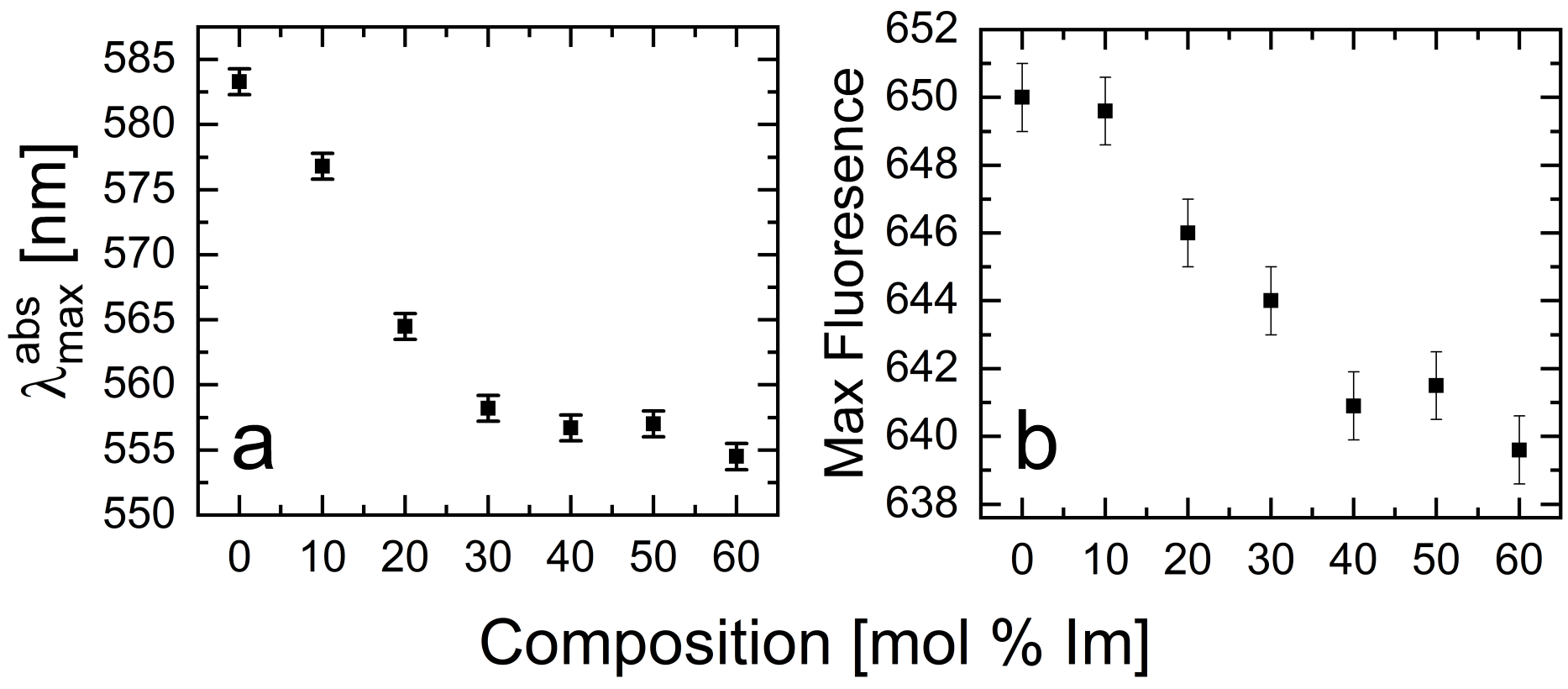

Figure S2 **a)** Maximum absorbance (λ_{max}^{abs}) and **b)** maximum emission (λ_{max}^{em}) wavelengths at 298 K. Nile Red (NR) is positively solvatochromic, meaning that solvent polarity increases as λ_{max}^{abs} and λ_{max}^{em} redshifts (increases). Stokes shift values in **Figure 3c** ($\Delta\lambda$, turquoise pentagons) are calculated as the difference in these values.

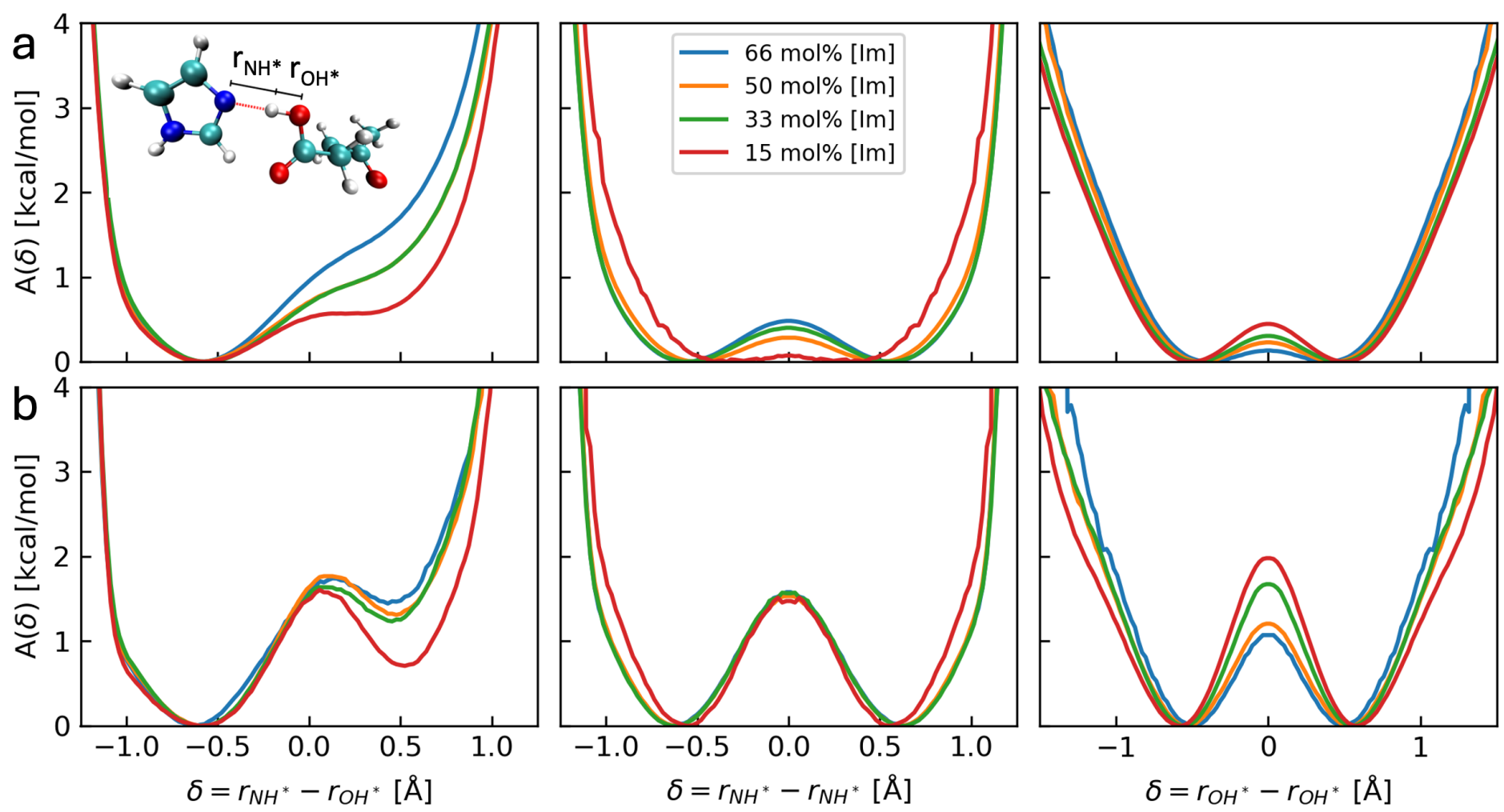

Figure S3 **a)** Free energy barriers along the proton sharing coordinate (δ) between imidazole and LA molecules (left) as sampled via PIMD simulations. Lower free energies for $\delta < 0$, corresponding to when H^* is covalently bound to imidazole, indicate that reactive protons are preferentially stabilized on imidazole. The proton transfer free energy barriers between two imidazole (middle) and two LA (right) molecules are also shown. **b)** The corresponding free energy barriers sampled via MD present overestimated reaction barriers as a result of not accounting for NQEs.

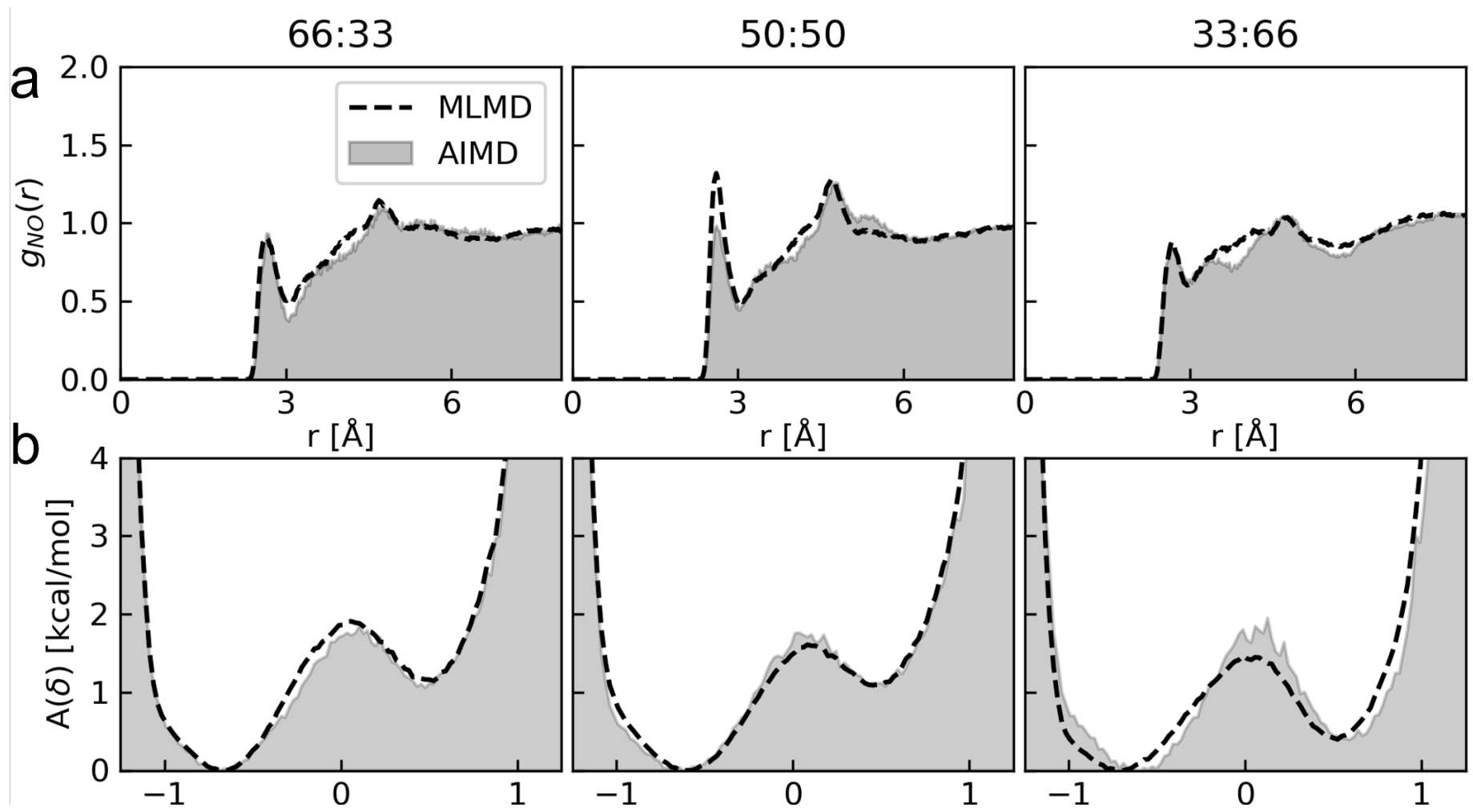

Figure S4 Benchmarking the accuracy of our ML potential energy surface model in reproducing static and reactive properties obtained from classical MD simulations. Results obtained from MD simulations employing our ML model (dotted lines) reproduced radial distribution functions (a) and proton transfer free energy barriers (b) obtained from reference AIMD simulations (shaded) for 66 mol% Im at 383 K run with DFT (PBE-D3). Benchmark simulations were run at elevated temperatures in order to appreciably sample proton transfers over the course of the short AIMD simulations (∼45 ps) in order to make a statistically meaningful comparison of free energy barriers. These validations demonstrate the accuracy of our ML model, which we then use as a proxy for DFT (PBE functional with D3 dispersion correction) in all other MD simulations reported in this work.

	a_1	a_2	τ_1 [ps]	τ_2 [ps]	τ_3 [ps]
15 mol% Im	0.11	0.07	0.2	10	1901
33 mol% Im	0.13	0.07	0.3	52	2283
50 mol% Im	0.13	0.10	0.3	39	2274
66 mol% Im	0.13	0.13	0.3	128	1026

Table S2 Tri-exponential fit parameters to the imidazole orientational time correlation functions. The tends in these timescales roughly follows the trends in viscosities. 66 mol% Im in particular shows a considerably faster imidazole reorientational timescale as compared to the other compositions.

	15 mol% Im	33 mol% Im	50 mol% Im	66 mol% Im
ML-MD	3.33	3.96	3.96	4.47
ML-PIMD	3.68	4.93	4.94	6.03

Table S3 Average hydrogen bonded chain lengths, in number of molecules, obtained from our ML-MD and ML-PIMD simulations. In neglecting NQEs, the chain lengths are considerably shorter for all compositions.

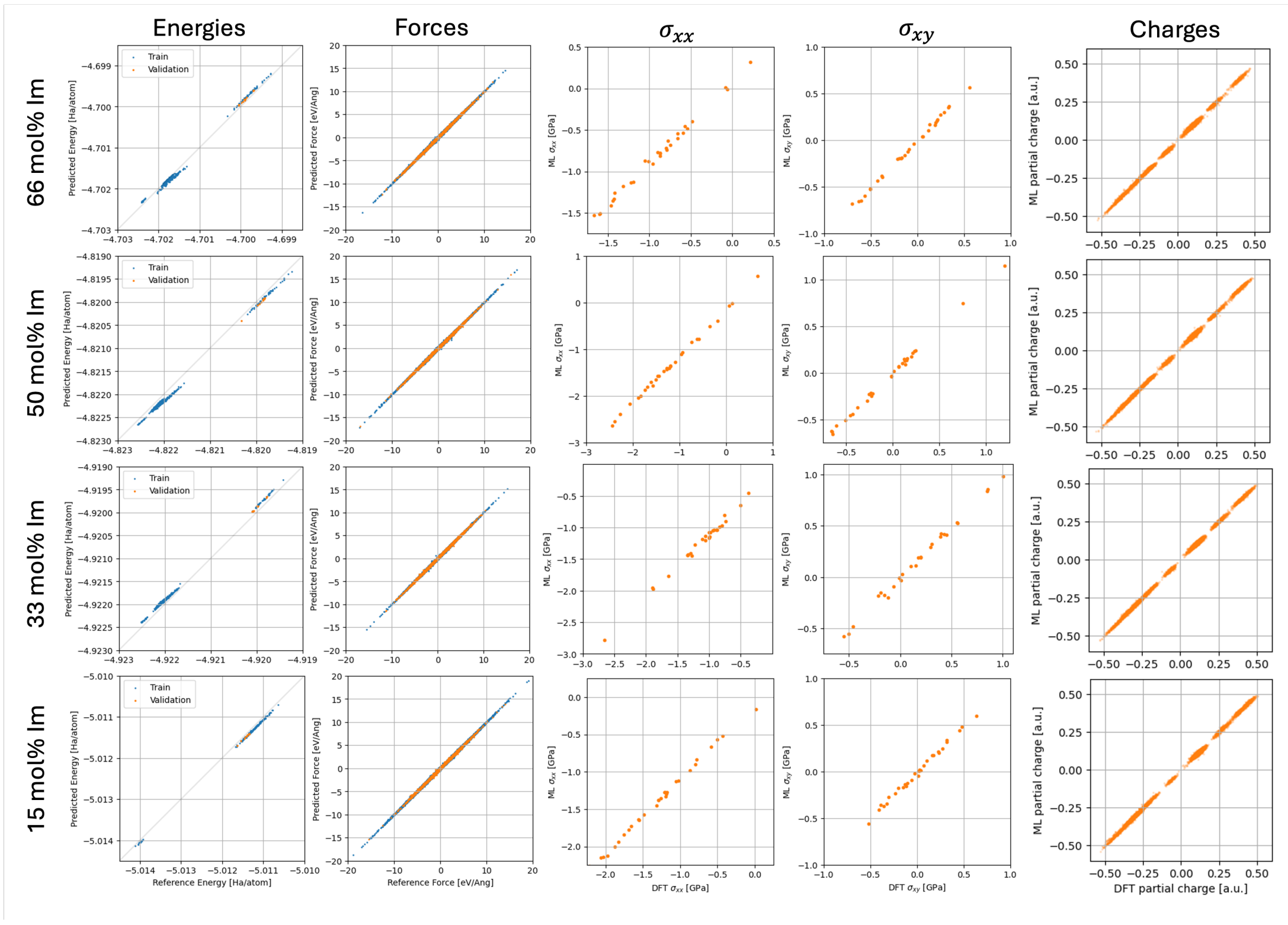

Figure S5 Benchmarking the prediction accuracy of our ML potential energy surface model and ML charge model in reproducing the reference DFT energies, atomic forces, stress tensors (both diagonal σ_{xx} and off-diagonal σ_{xy} components), and partial atomic charges that make up our validation set.

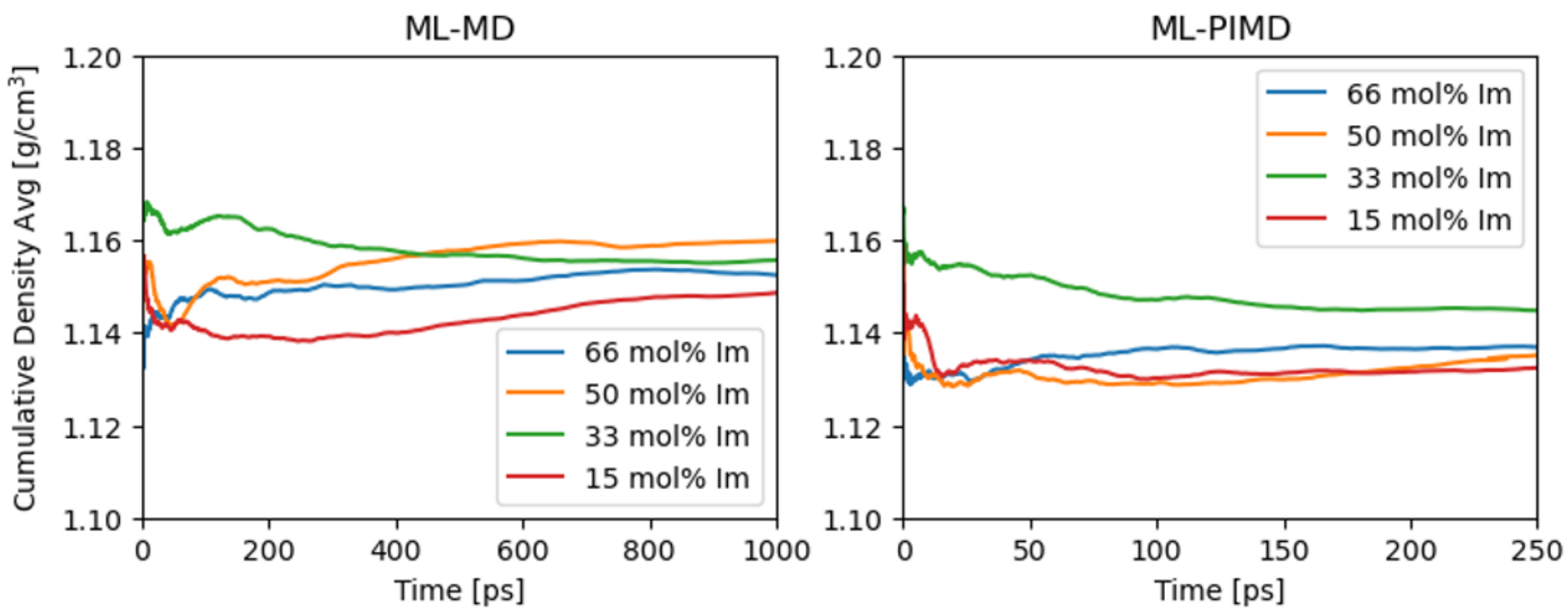

Figure S6 Tracking the cumulative density averages as a function of simulation time, for both our ML-MD (left) and ML-PIMD (right) simulations sampling the NPT ensemble, showing the long timescales required for the equilibration of the densities.

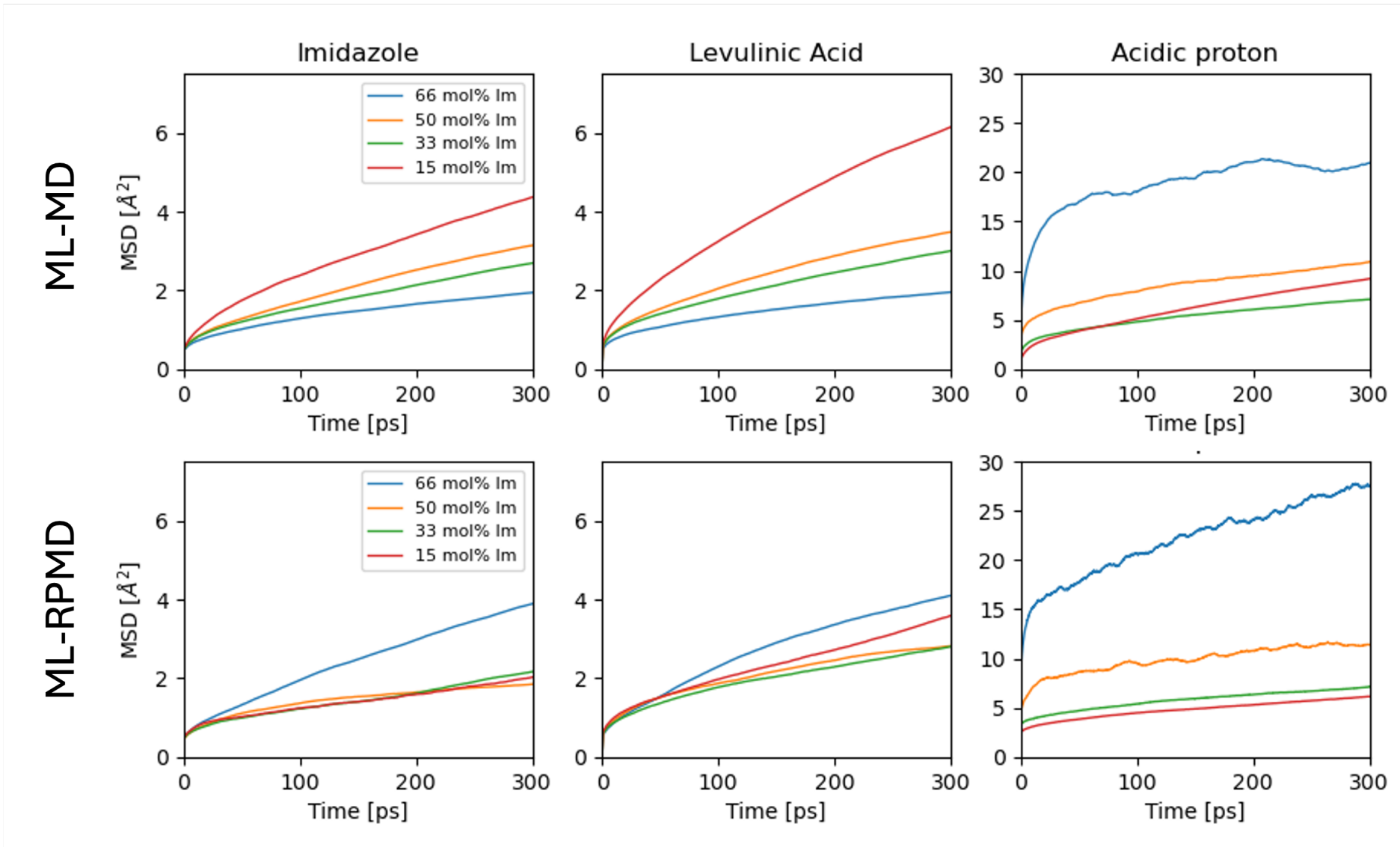

Figure S7 Mean square displacements computed for imidazole (left), levulinic acid (middle), and the acidic proton defects (right) for the different compositions as obtained from ML-MD (top) and ML-RPMD (bottom) simulations. Diffusion coefficients reported in this work were obtained from a linear fit of these MSDs from 200 to 300 ps.

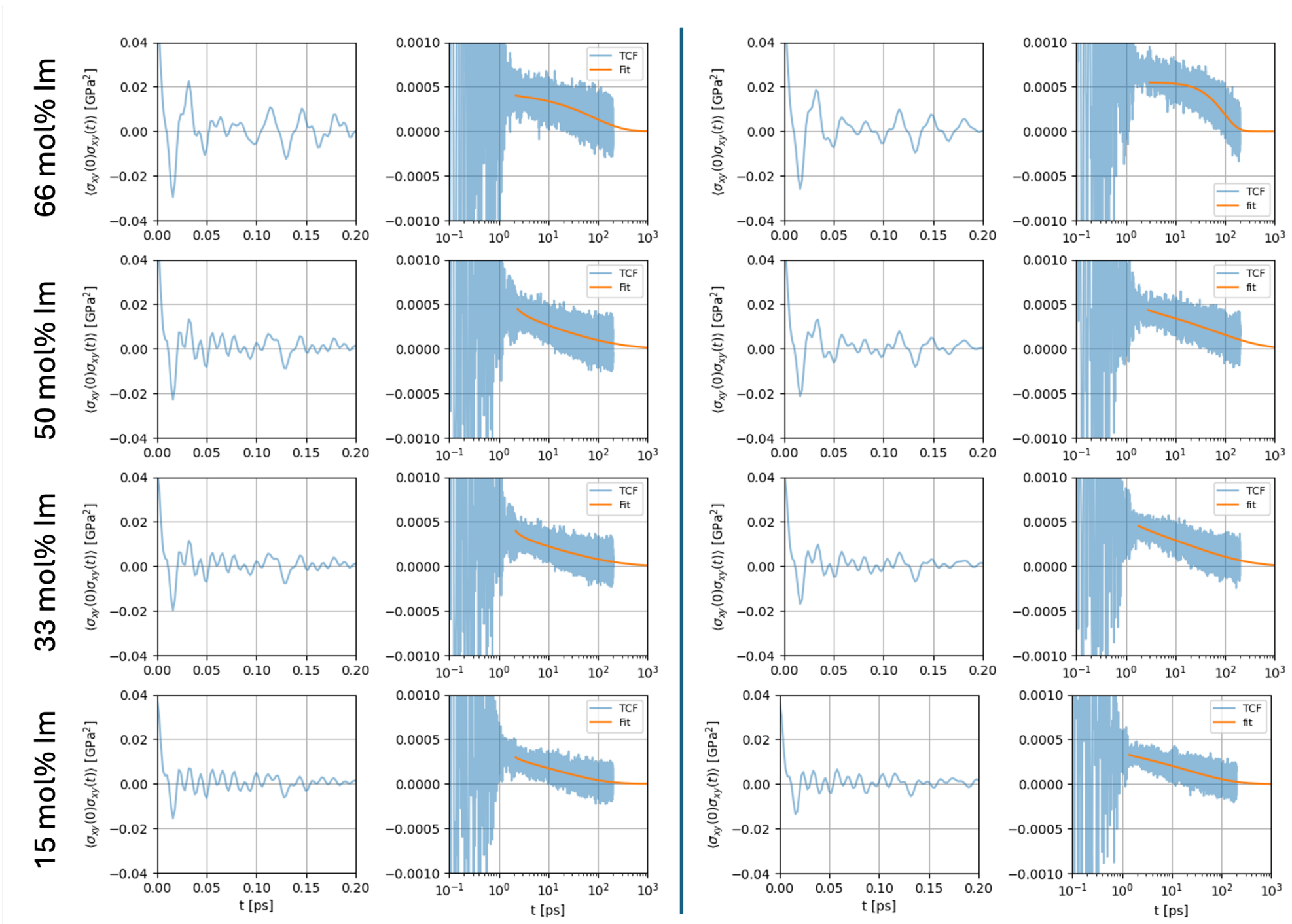

Figure S8 Stress tensor time correlation functions (TCF), as obtained from both ML-MD and ML-RPMD simulations, used to compute our reported viscosity estimates. A pair of plots is shown for each composition with the plots on the left showing the TCF with a linear x-scale ranging from 0 to 0.2 ps while the plots on the right show the same TCF (blue) on a log x-scale along with the fitted long-time decay (orange) past the cutoff time t_c=200 ps.

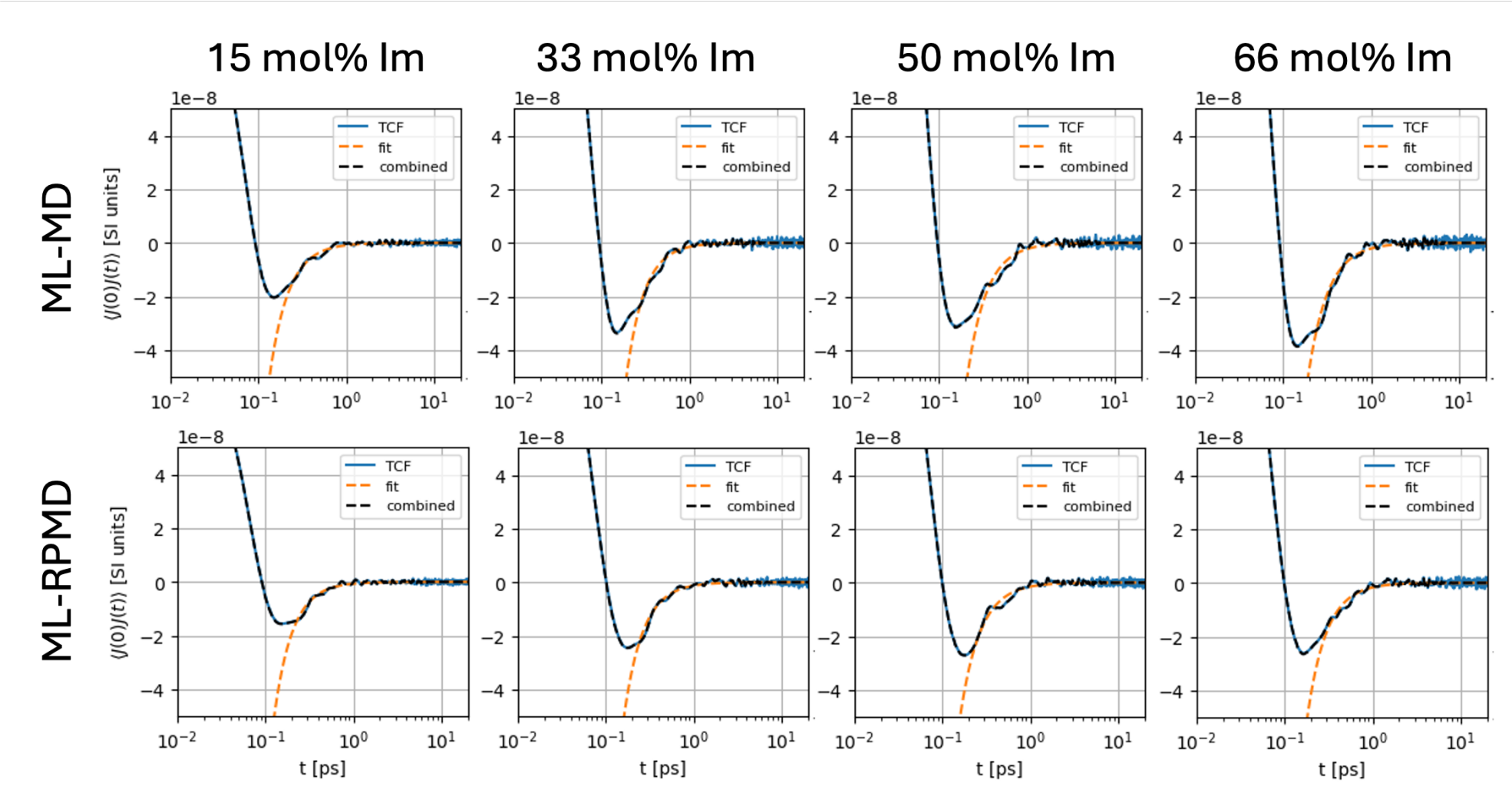

Figure S9 Electric current time correlation functions (TCF), as obtained from both ML-MD and ML-RPMD simulations, used to compute our reported conductivity estimates. The computed TCFs (blue line) are plotted on a a log x-scale along with the fitted long-time decay (orange) past the cutoff time t_c=5 ps. The black lines, which we integrate to obtain our conductivity estimates, are computed as a linear combination of the two whilst applying a switching function of the form $\tanh((t - t_c)/\tau + 1)/2$ with τ=1 ps.

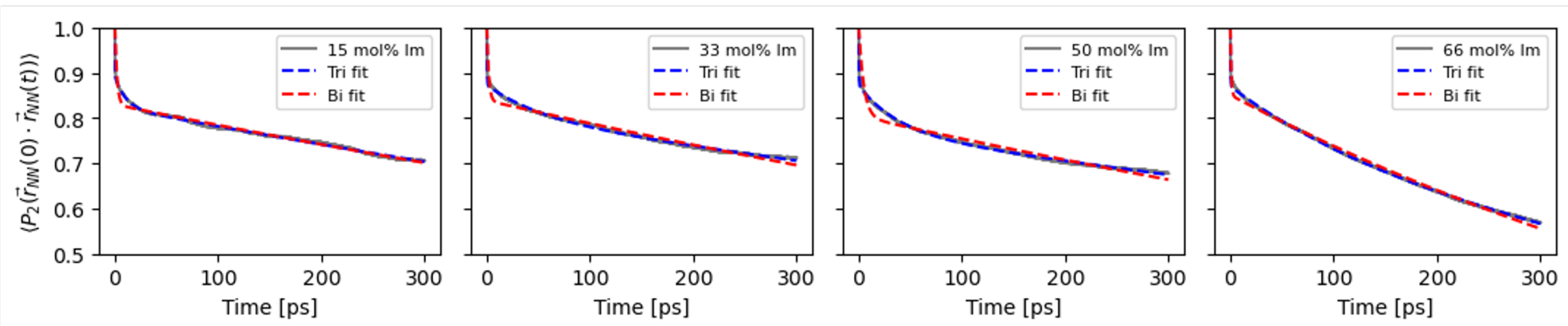

Figure S10 Orientational time correlation functions for imidazole (black line), based on the vector $\vec{r}_{NN}$ connecting a given imidazole molecule's nitrogen atoms, as obtained from our ML-RPMD simulations along with tri-exponential (blue lines) and bi-exponential fits (red lines) to these TCFs.

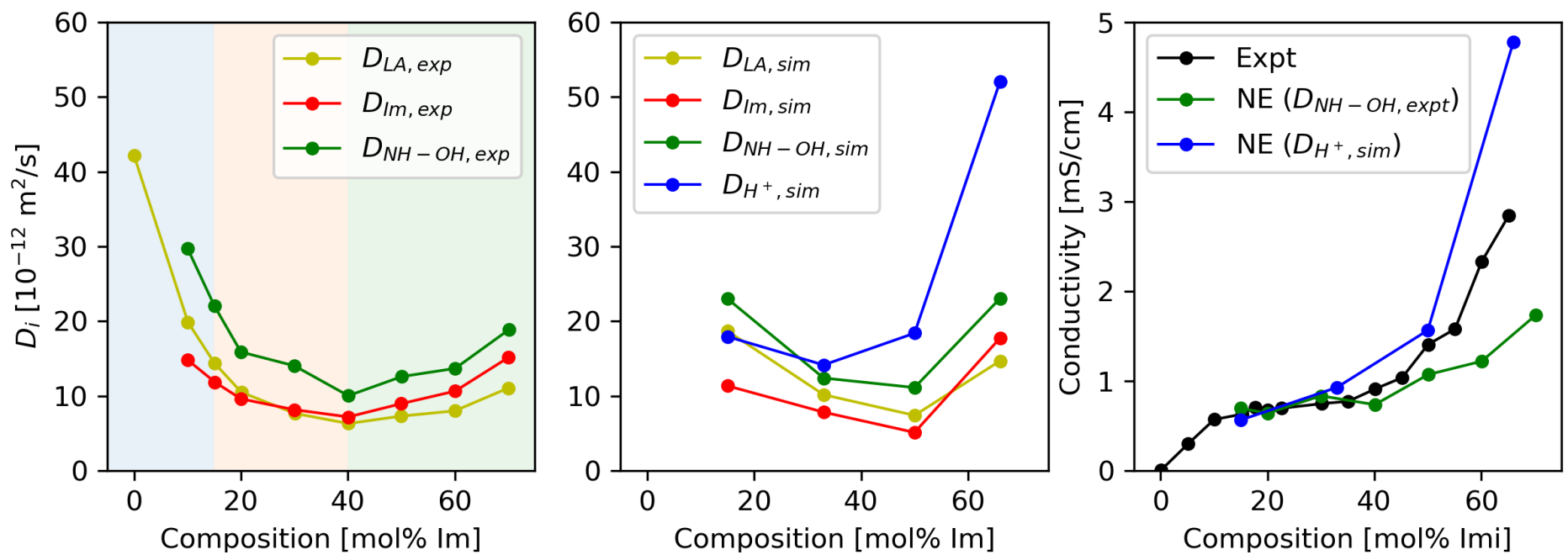

Figure S11 **(left)** Vehicular diffusion constants obtained from PFG-NMR for levulinic acid ($D_{LA,exp}$), imidazole ($D_{Im,exp}$), and acidic hydrogen atoms ($D_{NH-OH,exp}$). Slowing diffusion rates reach a local minimum around 40 mol% Im before increasing through Region III. $D_{Im,exp}$ and $D_{LA,exp}$ approach and crossover in Region II. Average diffusion rates for the acidic protons $D_{NH-OH,exp}$ are faster everywhere. **(middle)** Diffusion constants obtained from RPMD simulations qualitatively follow the experimental trends. From our simulations, we compute $D_{H^+,sim}$, which both tracks the diffusivity of acidic protons and additionally accounts for any diffusion via GM. **(right)** Nernst-Einstein conductivities estimated using $D_{NH-OH,exp}$ (green) and $D_{H^+,sim}$ (blue) as compared to the measured conductivities (black).
